# *FANCM* c5791C>T stopgain mutation (rs144567652) is a familial colorectal cancer risk factor

**DOI:** 10.1002/mgg3.1532

**Published:** 2020-10-29

**Authors:** Lisa A. Cannon‐Albright, Craig C. Teerlink, Jeffrey Stevens, Angela K. Snow, Bryony A. Thompson, Russell Bell, Kim N. Nguyen, Nykole R. Sargent, Wendy K. Kohlmann, Deborah W. Neklason, Sean V. Tavtigian

**Affiliations:** ^1^ Division of Epidemiology Department of Internal Medicine University of Utah School of Medicine Salt Lake City UT USA; ^2^ Huntsman Cancer Institute University of Utah Salt Lake City UT USA; ^3^ Department of Pathology Royal Melbourne Hospital Melbourne Australia; ^4^ Department of Oncological Sciences University of Utah School of Medicine Salt Lake City UT USA

**Keywords:** colorectal cancer, *FANCM*, high‐risk pedigree, UPDB

## Abstract

**Purpose:**

While familial aggregation of colorectal cancer (CRC) is recognized, the majority of the germline predisposition factors remain unidentified, and many high‐risk CRC pedigrees remain unexplained by known risk variants. Fanconi Anemia genes have been recognized to be associated with cancer risk. Notably, *FANCM* (OMIM 609644) variants have been reported to confer risk for CRC and breast cancer.

**Methods:**

Exome sequencing of CRC‐affected cousins in a set of 47 independent extended high‐risk CRC pedigrees identified a candidate set of rare, shared variants. Variants were tested for association with risk in 744 Utah CRC cases and 1525 controls, and for segregation with CRC in affected relatives.

**Results:**

A *FANCM* stopgain variant was observed in two CRC‐affected cousin pairs, each from an independent Utah high‐risk pedigree, and yielded a nonsignificant, but elevated OR = 2.05 in a set of Utah cases and controls. Segregation of the variant to other related CRC‐affected cases was observed in the two extended pedigrees.

**Conclusion:**

A rare stopgain variant in *FANCM* (rs144567652) that is recognized as a breast cancer predisposition variant, and that has previously been proposed, but not confirmed, as a CRC predisposition variant, is validated here as a risk factor for familial CRC.

## Graphical abstract

This manuscript reports a unique and efficient study design including high‐risk pedigrees to identify strong candidate predisposition variants for colorectal cancer. It validates a FANCM risk variant previously reported as associated with colorectal cancer, but not confirmed. These confirmatory findings include two segregating high‐risk pedigrees and provide information useful for clinical guidance for carriers with respect to cancer risk.

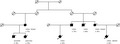

**Abbreviations**
CRC: colorectal cancer
OR: odds ratio

## 1 INTRODUCTION

Up to 30% of colorectal cancer (CRC) cases have a hereditary basis, with an estimated 5% of CRC occurring due to a genetically defined high‐risk CRC syndrome involving pathogenic germline mutations in *APC* (OMIM 611731), *MUTYH* (OMIM 604933), and DNA mismatch repair (MMR) genes (*MLH1* (OMIM 120436), *MSH2* (OMIM 609309), *MSH6* (OMIM 600678), *EPCAM* (OMIM 185535), and *PMS2* (OMIM 600259)). Other established but less prevalent CRC risk genes include *STK11* (OMIM 602216), *BMPR1A* (OMIM 601299), *GREM1* (OMIM 603054), *POLE* (OMIM 174762), *POLD1* (OMIM 174761), *NTHL1* (OMIM 602656), *MLH3* (OMIM 604395), *MSH3* (OMIM 600887), and *SMAD4* (OMIM 600993) (Seifert et al., 2019; Valle et al., 2019). Despite these multiple genes identified to be associated with CRC, many tested CRC families have no genetic diagnosis made. Identifying new genetic risk factors can contribute to our understanding of CRC susceptibility, initiation, and progression.

Fanconi anemia (FA) is a rare recessive genetic disorder causing congenital growth abnormalities, bone marrow failure, and cancer predisposition. An association of FA genes with breast and ovarian cancer predisposition has been proposed (Bogliolo & Surralles, 2015; Thompson et al., 2012), and has been confirmed for breast cancer (Peterlongo et al., 2015). Esteban‐Jurado et al., (2016) have proposed this gene may also play a role in genetic predisposition to CRC.

Identification of rare variants shared in pairs of CRC‐affected relatives from high‐risk CRC pedigrees offers a powerful approach for the identification of rare predisposition genes/variants that is complimentary to both candidate gene studies and GWAS. This approach was utilized here to validate a previously reported candidate variant in *FANCM* (OMIM 609644; refseq NG_007417.1) and to present additional candidates that should be further studied.

## 2 MATERIALS AND METHODS

### 2.1 Ethical compliance

University of Utah Institutional Review Board ethics approval was in place for all studies noted. Data from UPDB may not be shared publicly.

### 2.2 Utah high‐risk CRC pedigrees

Studies of high‐risk Utah CRC pedigrees have been performed in Utah since the 1950s (Burt et al., 1985; Cannon‐Albright et al., 1988; Neklason et al., 2008; Woolf et al., 1955). Germline DNA extracted from whole blood was collected for consenting cancer affecteds and relatives, but no tumor tissues were collected. Many of the pedigrees studied were originally identified in the Utah Population Database (UPDB), a computerized genealogy of Utah that includes original genealogy data from the Utah founders in the mid‐1800s. This database has been updated with vital statistics data for Utah to extend genealogy to modern day, and is linked to the statewide Utah Cancer Registry which was established in 1966. The Utah Cancer Registry became an NCI Surveillance, Epidemiology, and End Results (SEER) Registry in 1973 and is linked annually to the Utah genealogy in the UPDB. It registers all independent primary cancers diagnosed or treated in Utah, allowing identification of all clusters of related CRC cases (pedigrees).

Forty‐seven independent high‐risk CRC pedigrees, each containing a sampled CRC‐affected cousin pair, were selected from high‐risk pedigree studies performed in Utah over the last several decades. The majority of these pedigrees were validated as high‐risk by the observation of a significant excess of CRC cases among the descendants of the common ancestor of a set of related CRC cases (pedigree) using sex‐ and birth‐year cohort‐specific expected rates of CRC estimated internally in the UPDB (Cannon‐Albright, 2008).

### 2.3 Identification of candidate predisposition variants

Exome sequencing was performed on germline DNA extracted from whole blood from 94 CRC‐affected cases (47 CRC‐affected cousin pairs) selected from Utah high‐risk CRC pedigrees. Exome sequencing was performed at the Huntsman Cancer Institute's Genomics Core facility. Genomic DNA (500 ng–1 µg) was sheared using a Covaris S2 instrument (Covaris, Woburn, MA, United States). Barcoded libraries were prepared using the NEBNext DNA Library Prep Reagent Set for Illumina (NEB # E6000) according to the manufacturer's instructions. Library enrichment was done with the Roche SeqCap EZ Choice Library (cat# 06266339001) and the SeqCap EZ Reagent Kit Plus v2 (NimbleGen #06‐953‐247‐001) using the manufacturer's protocol. Individual libraries were combined into pools of 6‐8 prior to hybridization. Captured library pools were combined into groups of 21‐39 samples for sequencing on a HiSeq 2000 channel using the HiSeq 101 Cycle Paired‐End sequencing protocol. Exomes had an average of >50X coverage with 80% of targets having 30x coverage and <5% with zero coverage. Variant discovery from the sequencing data was conducted using the Broad Institutes’ Genome Analysis Tool Kit (GATK) Best Practices germline workflows (DePristo et al., 2011). Briefly, sequences were aligned to the human reference genome (GRCh37) using BWA (Li & Durbin, 2009); BAM file analyses and processing was conducted using Picard (http://broadinstitute.github.io/picard) and GATK; and joint variant calling was conducted using GATK’s HaplotypeCaller. VCFtools was used to conduct quality control to exclude bad quality samples and confirm relatedness in the CRC target pairs (Danecek et al., 2011). Variants occurring outside the exon capture kit intended area of coverage were removed. Candidate variants were annotated with ANNOVAR software (Wang et al., 2010). Candidate variants were filtered on the criteria of being rare in population (MAF < 0.005) and shared by an index cousin pair.

### 2.4 Case/control set

Germline DNA from whole blood was available for 597 CRC cases and 1590 Utah cancer‐free controls. Germline DNA from formalin‐fixed paraffin‐embedded (FFPE) grossly uninvolved tissue was available for an additional 177 Utah CRC cases. Cases were selected as individuals affected with CRC and either: (a) having a first‐degree relative with CRC, or (b) belonging to an extended high‐risk CRC pedigree. Cases were excluded for having a genetic diagnosis of a CRC predisposition syndrome, but not excluded for a *clinical* diagnosis with no prior genetic testing. Controls were defined as individuals lacking a diagnosis of any cancer in the Utah Cancer Registry and having no first‐degree relatives with any cancer and no second‐degree relatives with CRC (1353 controls) or having no first‐degree relatives with breast cancer or CRC (172 controls) in the UPDB. VCFtools was used for quality control and to exclude duplicated/related samples (Danecek et al., 2011). Samples in the case‐control cohort that had a heterozygosity score <−0.8; missing rate >10%; and mean depth <20 reads were excluded from further analysis. Individuals with a kinship coefficient (Manichaikul et al., 2010) >0.25 were investigated further for relatedness; 30 cases and 65 controls were excluded from the analysis because of sequencing failure or sample duplication, leaving 744 cases and 1525 controls.

### 2.5 Case/control validation

Genomic DNA (150 ng) was sheared using a Covaris E‐series instrument (Covaris). Libraries were prepared using the Ovation Ultralow Library System v2 (NUGEN # 0347‐ A01). For DNA extracted from FFPE, 2 µl of Uracil‐DNA Glycosylase (UDG) (NEB #M0280S) were added to samples following adapter ligation and incubated for 37°C for 15 min, prior to ligation cleanup in order to reduce C>T artifacts (Do & Dobrovic, 2012). A custom CRC gene panel consisting of 197 genes was created, and the 744 cases and 1525 cases and controls were assayed. This panel included 59 known/candidate cancer susceptibility genes identified through literature review, and 138 additional genes selected from the various gene prioritization analyses using PERCH (Feng, 2017) and p‐VAAST (Hu et al., 2014) (data not shown). This was independent of the study described here, and did not include all of the variants identified as rare variants shared in the CRC‐affected cousin pairs. Library enrichment for the custom CRC gene panel was done with the Roche SeqCap EZ Choice Library (cat# 06266339001) and the SeqCap EZ Reagent Kit Plus v2 (NimbleGen #06‐953‐247‐001) using the manufacturer's protocol. Individual libraries were combined into pools of 12 prior to hybridization, and then, super‐pooled into 72 samples per sequencing lane. Captured libraries were sequenced on an Illumina HiSeq 2000 channel using the HiSeq 101 Cycle Paired‐End sequencing protocol. Variant discovery for the case/control sequencing used the GATK Best Practices germline workflows as above (DePristo et al., 2011). A two‐tailed Fisher's exact test (R software) was used for case‐control association tests. Because the focus of this study was rare variants (MAF < 0.005) which were observed to be carried by both CRC‐affected cousins in a high‐risk CRC pedigree, there was not sufficient power in the case/control data set to identify significant association with CRC risk. Instead, we identified rare shared candidate variants that exhibited an estimated OR > 2.0, and analyzed them for segregation in the high‐risk pedigrees in which they were originally observed.

### 2.6 Validation of variant segregation in high‐risk pedigrees and relatives

The 47 high‐risk CRC pedigrees from which the original CRC‐affected cousin pairs were selected typically included additional sampled individuals, including CRC‐affected relatives. Sampled relatives of the two CRC‐affected variant‐carrying cousin pairs were assayed (TaqMan assay # C_163079670_10) for the variant, to determine segregation with CRC. Assay of the variant was also performed for all sampled relatives of the two singleton CRC case carriers identified (from the 744 cases) in the case/control validation study. The RVsharing program (Bureau et al., 2014) was used to assess the probability of the observed configuration of sharing in pedigrees where multiple CRC cases were found to carry the rare variant allele. RVsharing provides an exact probability expressed as a *p* value and assumes the shared variant is rare (<1%) and has entered the pedigree once.

## 3 RESULTS

Bioinformatic analysis identified 869 rare (MAF < 0.005) shared candidate coding variants in 767 genes that were observed in at least one pair of CRC‐affected cousins from the 47 independent high‐risk pedigrees. This entire set of 869 candidate CRC predisposition variants is listed in Table S1. Of these variants, 330 variants were located in one of the 197 genes identified from the various candidate gene prioritization analyses or from literature review that were independently tested on the case/control set, and were therefore, included in the case/control validation analysis for this study. Odds Ratios (OR) were estimated for these 330 variants in the 744 Utah cases and 1525 Utah controls. ORs > 2.00 were observed for six candidate variants (Table 1). Of additional interest are six rare candidate variants that were observed in at least one CRC case but in no controls (Table 2).

**TABLE 1:** Rare candidate variants observed shared in affected cousins from high‐risk pedigrees, and with OR>2.0 in Utah case/control association.

*Gene (OMIM)*	GRCh37 (chr:pos)	Variant rsID	GnomAD2.2 frequency	OR	Exonic function
*CEP350* (617870)	(1:179959750)	rs140306241	0.0035	2.22	nonsynonymous SNV
*BARD1* (601593)	(2:215593618)	rs149262370	0.0001	2.03	nonsynonymous SNV
*FRAS1* (607830)	(4:79432453)	rs186811333	0.00096	2.84	nonsynonymous SNV
*TNXB* (600985)	(6:320225841)	rs757463918	0.00001	4.14	nonsynonymous SNV
*FANCM* ^a^ (60964)	(14:45667921)	rs144567652	0.001	2.05	stopgain
*YLPM1* (NA)	(14:75266265)	rs369089869	0.000124	2.02	nonsynonymous SNV

^a^ refseq NG_007417.1.

**TABLE 2:** Rare candidate variants observed shared in affected cousins from high‐risk pedigrees, and in at least one validation CRC case, but in no Utah validation controls.

Gene (OMIM)	GRCh37 (chr:pos)	Variant rsID	GnomAD2.2 frequency	OR	Exonic function
*SYNE2* (608442)	(14:64518503)	rs140325055	0.00048	NA	Nonsynonymous SNV
*SYNE2* (“)	(14:64554434)	rs140243093	0.00041	NA	Nonsynonymous SNV
*ZFYVE26* (612012)	(14:68229060)	rs140540720	0.0017	NA	Nonsynonymous SNV
*MLH3* (604395)	(14:75516351)	rs114829239	0.0023	NA	Nonsynonymous SNV
*XYLT1* (608124)	(16:17232375)	rs199714091	0.0002	NA	Nonsynonymous SNV
*USHBP1* (611810)	(19:17373761)	rs141014235	0.00001	NA	Nonsynonymous SNV

Of the six candidate variants with OR >2.00, *FANCM* c.5791C>T (rs144567652) was the only variant which was observed in more than one affected‐cousin pair; this variant has also been previously published as associated with CRC risk (Smith et al., 2013). The *FANCM* variant was observed in 2/744 Utah cases and in 2/1525 controls (OR = 2.05, Fisher's exact two‐tailed *p* = .60). The *FANCM* variant was not observed in any CRC cases in two external public data sets (DbGap phs001554.v1.p1: 0/3,854 cases, UK biobank: 0/6,426 cases).

**FIGURE 1:**
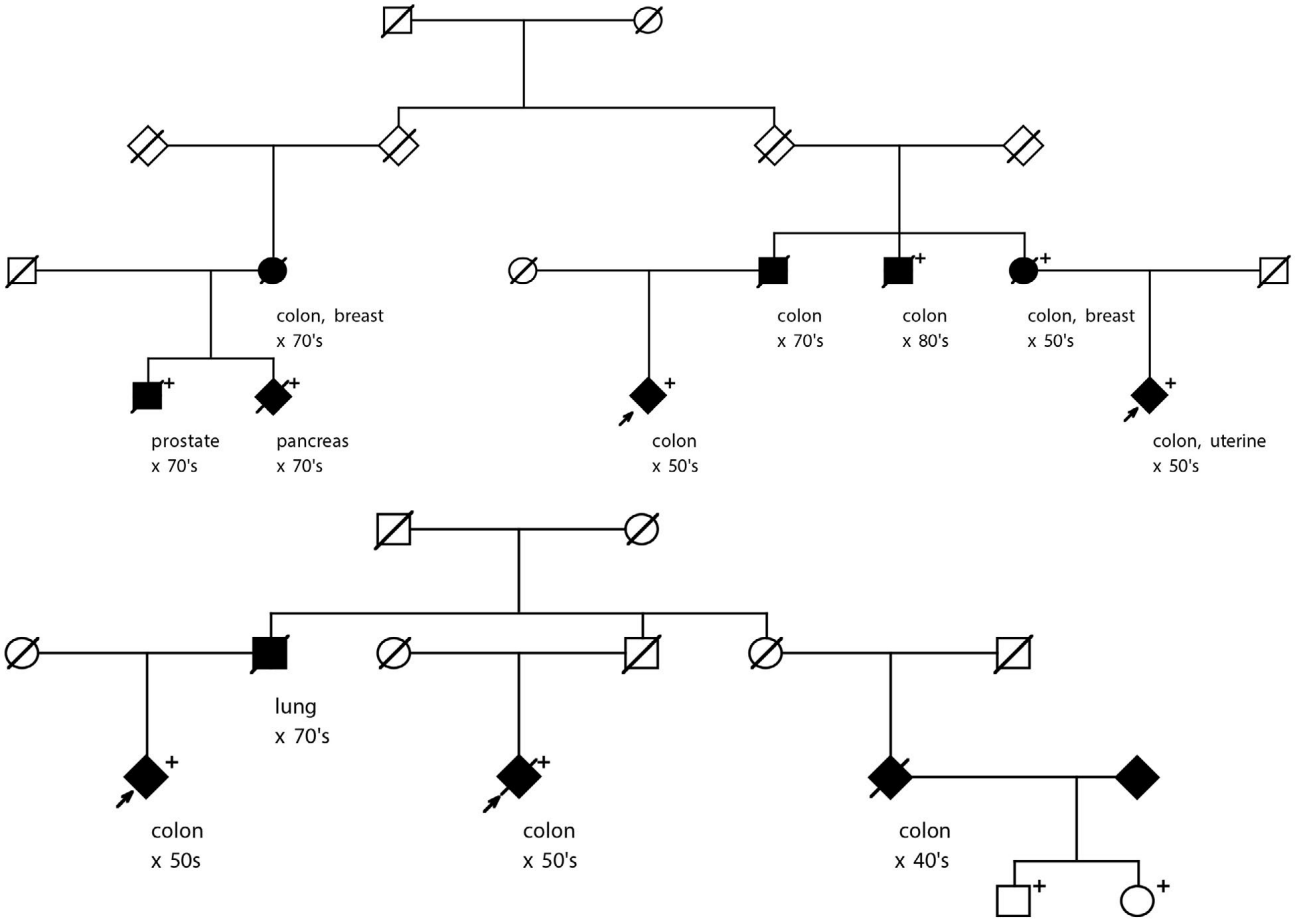
Segregation of rare *FANCM* rs144567652 stopgain variant in two high‐risk pedigrees in which it was originally identified as shared in affected cousins. The original CRC‐affected cousin pair is shown with arrows. Fully shaded =cancer diagnosis, age at diagnosis is shown by decade; “+” indicates assayed/confirmed carrier of rs144567652 variant; all other individuals shown did not have samples available for screening.

Segregation of the *FANCM* candidate variant was analyzed in the two high‐risk pedigrees in which the variant was originally observed in an affected‐cousin pair (Figure 1). The *FANCM* variant segregated to additional CRC cases in both pedigrees (RVsharing *p* = 0.012; *p* = 0.002, respectively). All of the CRC cases assayed in the two pedigrees carry the rare *FANCM* variant, as do some cases of other cancers, including lung, breast, prostate, pancreas, and uterine cancer cases. All of the CRC cases in the two pedigrees are colon cancers (no rectal cancers observed). The average age of the colon cancers in the first pedigrees is young (mean = 51 years), but is not young in the second pedigree (mean = 69 years).

In the case/control association study, two additional CRC case carriers were identified. The first case was diagnosed in their 60 s and had five CRC‐affected siblings, four of whom were assayed for the variant, and two of whom were carriers. The second case carrier was diagnosed in their 50 s and had a parent diagnosed with colon cancer in their 70 s; no DNA for any relatives was available for testing. With respect to our observation of two controls with rs144567652, we note that our selection criterion for controls included lack of family history of CRC; consequently, exploring their pedigrees would be pointless.

## 4 DISCUSSION

We performed exome sequencing in germline DNA from CRC‐affected cousin pairs from 47 sampled high‐risk CRC pedigrees, and identified 869 rare (MAF < 0.005) candidate CRC predisposition variants. A subset of these candidate variants had been previously screened in 744 Utah CRC cases and 1525 cancer‐free controls to establish association with CRC risk. No candidate variants achieved significant association with CRC status in the Utah cases and controls, as expected due to the low power of this study design to identify rare variants. This study instead focused on the effect size of rare variants shared in CRC‐affected relatives from high‐risk pedigrees, noting those that achieved an OR > 2.0 in case/control association, regardless of significance. A set of 12 strong candidate variants was identified: six variants showed OR ≥ 2.0 for CRC risk association (Table 1), and six variants were observed in cases but no controls (Table 2). A rare variant in *FANCM*, exhibiting OR = 2.05 in the case/control set was observed in two independent sets of CRC‐affected cousins from high‐risk pedigrees. The observation of segregation of this variant to multiple CRC cases in multiple CRC pedigrees adds to existing evidence and validates the *FANCM* c5791C>T stopgain mutation (rs144567652) as a pathogenic variant for CRC.

Analysis of affected individuals belonging to high‐risk pedigrees has long been a powerful design for identification of rare cancer predisposition genes and variants in Utah (e.g., *BRCA1* [OMIM 113705], *BRCA2* [OMIM 600185], *CDKN2A* [OMIM 600160], *GOLM1* [OMIM 606804], *APC*, *PTEN* [OMIM 601728]). Of course, external validation should be established before a variant is considered to be associated with increased risk; some proposed variants for CRC have not been validated to date (Broderick et al., 2017). While pedigree studies are powerful for identification of the rarest of pathogenic variants, subsequent validation in a large case/control study is often hampered by low power for the rare variants. Here, we follow‐up on a sequencing study of pairs of CRC‐affected relatives from high‐risk pedigrees which identified rare candidate variants shared in at least one affected pair from a high‐risk pedigree (see Table S1 for the entire list of candidates). As expected based on the sample sizes, none of these rare candidates were validated in the small Utah case/control analysis attempted (744 cases, 1525 controls). Some candidates yielded OR ≥ 2.0 (Table 1) or were observed in independent cases, but not in controls (Table 2); all of these candidates deserve further consideration. A rare variant in *FANCM* was observed in cousins in two high‐risk pedigrees; has previously been reported as a breast cancer predisposition variant; has been reported (Smith et al., 2013), but not confirmed (Broderick et al., 2017) as associated with CRC risk, and has been analyzed further here.

Fanconi Anemia‐related genes are good cancer candidates. A link between heterozygous pathogenic variants in Fanconi Anemia genes and cancer predisposition has been suggested for breast and ovarian cancers (Bogliolo & Surralles, 2015; Thompson et al., 2012). In an analysis of exome sequence data of 74 CRC patients from high‐risk pedigrees, Esteban‐Jurado et al., (2016) noted an enrichment for variants in Fanconi Anemia DNA damage repair pathway genes, and concluded the Fanconi Anemia DNA damage repair pathway might also play an important role in heritable predisposition to CRC. In an analysis of the transcriptome of a cell line derived from metastasis‐competent circulating tumor cells of a patient with colon cancer, Alix‐Panabieres et al., (2017) identified *FANCM* as one of several genes involved in colon cancer progression.

The *FANCM* c.5791C>T variant (rs144567652) is predicted to introduce a stop codon (TGA) in exon 22 (p.Arg1931Ter), causing the loss of 118 amino acids from the C‐terminal end of the main transcript and putatively influencing *FANCD2* (OMIM 613984) monoubiquitination (Gracia‐Aznarez et al., 2013). Peterlongo et al. (2015) established that *FANCM* rs144567652 also caused an out‐of‐frame deletion of exon 22 in some transcripts due to the creation of a binding site for the pre‐mRNA processing protein hnRNP A1, which is predicted to produce an alternative truncated protein p.Gly1906Alafs12Ter; genetic complementation analyses further showed that both truncated FANCM proteins had impaired DNA repair activity. [Correction added after first online publication: The in‐text citation Esteban‐Jurado et al. (2016) has been changed to Peterlongo et al. (2015).] Wang et al., (2018) showed a novel function of Fanconi anemia protein FANCM in the protection of common fragile sites that is independent of the Fanconi anemia core complex and the FANCI‐FANCD2 complex. ClinVar includes multiple submissions classifying rs144567652 as pathogenic.

Evidence for the association of the *FANCM* c.5791C>T variant (rs144567652) with CRC risk has been conflicting. Following a report of the *FANCM* rs144567652in a single proband from a high‐risk breast cancer pedigree (Gracia‐Aznarez et al., 2013; Peterlongo et al., 2015) reported that *FANCM* rs144567652 induces exon skipping, affects DNA repair activity, and is associated with familial breast cancer (OR =3.93) [Correction added after first online publication: The in‐text citation Esteban‐Jurado et al. (2016) has been changed to Peterlongo et al. (2015).]. Smith et al., (2013) reported *FANCM* rs144567652 in two sporadic patients with advanced CRC; tumors showed loss of the wild‐type allele. Broderick et al., (2017) identified additional case carriers of the variant; meta‐analysis of their data combined with two UK series provided no evidence for an association of the variant with CRC risk (*p* = 0.22). Here, we have provided independent replication of *FANCM* c.5791C>T, p.Arg1931Ter (rs144567652) and suggest that this variant predisposes to CRC and may explain some observed high‐risk CRC pedigrees.

Strengths of this study include the high‐quality diagnoses of all cancer cases present in the Utah NCI SEER Cancer Registry, the extensive genealogy data available, and the powerful study design allowing identification of rare candidate predisposition variants in CRC‐affected cousins who are members of high‐risk CRC pedigrees. The existence of confirmed cancer cases linked to extensive genealogy reduced the typical family‐study biases of ascertainment and recall. This study has again enforced the importance of high‐risk pedigrees for identification and validation of rare predisposition variants. Limitations of the study include the small case/control sample sizes, the potential for survival bias in the set of sampled CRC cases, and the censoring of relatives (affected and not) who were not captured in the Utah genealogy data or the Utah Cancer Registry data that results from both geographical and time limitations on UPDB data. These limitations may affect power and may inflate odds ratio estimates compared to population‐based studies, but do not introduce bias to the study.

The identification of this variant in two CRC‐affected cousin pairs in two independent high‐risk CRC pedigrees, and the significant evidence for segregation of the variant with CRC in additional relatives of these carriers adds to previous data to confirm the *FANCM* c.5791C>T stopgain mutation (rs144567652) as a familial colorectal cancer risk factor. Cancers of other sites observed in variant carriers suggests the variant may play a role in predisposition to cancer of other sites as well; further study of the pedigrees reported here, and of other variant carriers will clarify risks. The rare variants reported in Table 1, and those listed in Table S1 are all presented as strong candidate CRC predisposition variants that should be pursued as they are observed.

## CONFLICT OF INTEREST

The authors have no conflict of interest or commercial association to disclose.

## AUTHOR CONTRIBUTION

Lisa A. Cannon‐Albright, Craig C. Teerlink, Jeffrey Stevens, Angela K. Snow, Bryony A. Thompson, Russell Bell, Kim N. Nguyen, Nykole R. Sargent, Wendy K Kohlmann, Deborah W. Neklason, and Sean V. Tavtigian have all made substantial contributions to conception and design, acquisition of data, or analysis and interpretation of data. All authors were involved in drafting the manuscript or critical revision. All authors have provided final approval and agreed to be accountable for all aspects of the work.

## Supporting information

Supplementary Material

## Data Availability

The data that support the findings of this study are available on request from the corresponding author. The data are not publicly available due to privacy or ethical restrictions.
