# Supplementary material for: FANCM c5791C>T stopgain mutation (rs144567652) is a familial colorectal cancer risk factor
Source: Mol Genet Genomic Med. 2020 Oct 29;8(12):e1532. doi: 10.1002/mgg3.1532 (PMC7767553; doi:10.1002/mgg3.1532)
Supplement: Supplementary file 1 — Supplementary Material [file MGG3-8-e1532-s001.docx]

| **RefGene** | **Chr** | **Start** | **End** | **Ref** | **Alt** | **Func.refGene** | **ExonicFunc.refGene** | **Gnomad_AF** | **avsnp150** |
| --- | --- | --- | --- | --- | --- | --- | --- | --- | --- |
| CHD5 | 1 | 6203883 | 6203883 | G | A | exonic | synonymousSNV | 0.0004 | rs143956519 |
| CAMTA1 | 1 | 7829238 | 7829238 | C | T | UTR3 | . | 0.0022 | rs538077733 |
| SLC45A1 | 1 | 8390333 | 8390333 | G | A | exonic | synonymousSNV | 0.00006384 | rs768998199 |
| ENO1 | 1 | 8930951 | 8930951 | C | A | UTR5 | . | 0.0003 | rs557319521 |
| CTNNBIP1 | 1 | 9910659 | 9910659 | G | A | UTR3 | . | 0.0255 | rs71643073 |
| ANGPTL7 | 1 | 11254610 | 11254610 | T | C | exonic | synonymousSNV | . | rs748666510 |
| UBIAD1 | 1 | 11346336 | 11346336 | A | G | UTR3 | . | 0.023 | rs11580061 |
| C1orf167 | 1 | 11845272 | 11845272 | C | T | exonic | nonsynonymousSNV | 0.0022 | rs80132110 |
| MTHFR | 1 | 11847741 | 11847742 | CT | - | UTR3 | . | 0.0212 | rs35562212 |
| MTHFR | 1 | 11848670 | 11848670 | G | A | UTR3 | . | 0.0025 | rs149055513 |
| ARHGEF19 | 1 | 16525657 | 16525657 | A | G | exonic | nonsynonymousSNV | 0.0099 | rs148325785 |
| NBPF1 | 1 | 16890155 | 16890155 | C | T | UTR3 | . | 0.008 | rs1043520 |
| NBPF1 | 1 | 16910181 | 16910181 | C | G | exonic | unknown | 0.027 | rs201327573 |
| NBPF1 | 1 | 16915405 | 16915405 | G | C | exonic | unknown | 0.308 | rs201355992 |
| MST1L | 1 | 17083374 | 17083374 | A | C | UTR3 | . | 0.0864 | rs1759200 |
| EMC1 | 1 | 19564513 | 19564513 | G | A | exonic | nonsynonymousSNV | 0.00003184 | rs144713581 |
| TMCO4 | 1 | 20107163 | 20107163 | G | A | exonic | nonsynonymousSNV | . | rs780573948 |
| ZBTB40 | 1 | 22838561 | 22838563 | AAG | - | exonic | nonframeshiftdeletion | 0.0002 | rs779648270 |
| SH3BGRL3 | 1 | 26606488 | 26606488 | C | G | UTR5 | . | 0.0036 | rs529487179 |
| AIM1L | 1 | 26665898 | 26665898 | C | G | exonic | nonsynonymousSNV | . | . |
| AHDC1 | 1 | 27861172 | 27861172 | T | - | UTR3 | . | 0.0084 | rs1036538051 |
| STX12 | 1 | 28150034 | 28150034 | G | A | UTR3 | . | 0.0001 | rs751267476 |
| FNDC5 | 1 | 33330043 | 33330043 | T | - | UTR3 | . | 0.0112 | . |
| GJB5 | 1 | 35223884 | 35223884 | C | T | UTR3 | . | 0.0041 | rs545484486 |
| POU3F1 | 1 | 38509676 | 38509676 | G | C | UTR3 | . | 0.0052 | rs41268251 |
| POU3F1 | 1 | 38510322 | 38510322 | A | G | UTR3 | . | 0.0047 | rs186256033 |
| SMAP2 | 1 | 40888570 | 40888571 | GT | - | UTR3 | . | 0.00006445 | rs752902335 |
| PTPRF | 1 | 44088540 | 44088540 | T | A | UTR3 | . | 0.0002 | rs1130522 |
| ELAVL4 | 1 | 50574697 | 50574697 | A | C | UTR5 | . | 0.3977 | rs901208978 |
| EPS15 | 1 | 51887719 | 51887719 | - | A | UTR5 | . | . | . |
| RBMXL1 | 1 | 89448360 | 89448360 | C | T | exonic | nonsynonymousSNV | 0.0004 | rs146375168 |
| GBP5 | 1 | 89726438 | 89726438 | G | A | exonic | synonymousSNV | 0.0005 | rs148301962 |
| GBP6 | 1 | 89835209 | 89835209 | G | A | exonic | nonsynonymousSNV | 0.0108 | rs75966734 |
| ZNF644 | 1 | 91381239 | 91381239 | C | T | UTR3 | . | 0.0003 | rs529569741 |
| EXTL2 | 1 | 101338118 | 101338118 | - | A | UTR3 | . | 0.0087 | rs879127824 |
| CEPT1 | 1 | 111702020 | 111702020 | A | G | exonic | nonsynonymousSNV | 0.0103 | rs146158422 |
| TTF2 | 1 | 117635401 | 117635401 | G | A | exonic | nonsynonymousSNV | 0.0059 | rs41306197 |
| NOTCH2 | 1 | 120612297 | 120612297 | C | A | UTR5 | . | 0.1434 | rs11487653 |
| NBPF8,NBPF9 | 1 | 144828652 | 144828652 | C | T | exonic | unknown | 0.0026 | rs587753379 |
| PDE4DIP | 1 | 144915568 | 144915568 | A | T | exonic | synonymousSNV | 0.0053 | rs143827095 |
| NBPF9 | 1 | 145368472 | 145368472 | C | T | exonic | unknown | 0.0068 | rs201192694 |
| GPR89A | 1 | 145827011 | 145827011 | G | A | UTR5 | . | . | . |
| BCL9 | 1 | 147083650 | 147083650 | C | T | exonic | synonymousSNV | 0.0005 | rs41299583 |
| ENSA | 1 | 150598965 | 150598965 | G | A | exonic | nonsynonymousSNV | 0.0044 | rs145743127 |
| TCHH | 1 | 152081362 | 152081362 | C | G | exonic | nonsynonymousSNV | 0.0028 | rs182615256 |
| HRNR | 1 | 152187902 | 152187902 | T | C | exonic | nonsynonymousSNV | 0.0011 | rs201561609 |
| HRNR | 1 | 152187904 | 152187904 | G | A | exonic | synonymousSNV | 0.0019 | rs567251371 |
| HRNR | 1 | 152187906 | 152187906 | C | T | exonic | nonsynonymousSNV | 0.0028 | rs200036490 |
| HRNR | 1 | 152187907 | 152187907 | T | A | exonic | synonymousSNV | 0.0032 | rs549896192 |
| FLG | 1 | 152285861 | 152285861 | G | A | exonic | stopgain | 0.0096 | rs61816761 |
| PGLYRP3 | 1 | 153279596 | 153279596 | C | T | exonic | nonsynonymousSNV | 0.002 | rs148550417 |
| TDRD10 | 1 | 154516983 | 154516983 | G | A | exonic | nonsynonymousSNV | 0.0007 | rs146616270 |
| ASH1L | 1 | 155306064 | 155306064 | A | G | UTR3 | . | 0.0174 | rs116580423 |
| IQGAP3 | 1 | 156532932 | 156532932 | C | T | exonic | synonymousSNV | 0.0037 | rs76102113 |
| IQGAP3 | 1 | 156533433 | 156533433 | G | A | exonic | synonymousSNV | 0.0005 | rs75110454 |
| OR6Y1 | 1 | 158516967 | 158516967 | A | G | exonic | nonsynonymousSNV | 0.002 | rs143089565 |
| CFAP45 | 1 | 159863011 | 159863011 | C | T | exonic | nonsynonymousSNV | 0.0006 | rs143593292 |
| COPA | 1 | 160283802 | 160283802 | G | T | exonic | synonymousSNV | 0.00006379 | rs764849788 |
| NHLH1 | 1 | 160341110 | 160341110 | G | A | UTR3 | . | 0.0184 | rs114483127 |
| NIT1 | 1 | 161088292 | 161088292 | A | G | exonic | nonsynonymousSNV | 0.0049 | rs138523655 |
| TOMM40L | 1 | 161200380 | 161200380 | - | A | UTR3 | . | . | . |
| CACYBP | 1 | 174969202 | 174969202 | A | G | UTR5 | . | 0.0009 | rs755859164 |
| NPHS2 | 1 | 179544913 | 179544913 | G | C | exonic | synonymousSNV | 0.005 | rs12123397 |
| TDRD5 | 1 | 179599985 | 179599985 | C | T | exonic | synonymousSNV | 0.0051 | rs12126190 |
| TOR1AIP2 | 1 | 179833802 | 179833802 | A | T | UTR3 | . | 0.0001 | rs752180502 |
| CEP350 | 1 | 179959750 | 179959750 | A | G | exonic | nonsynonymousSNV | 0.0031 | rs140306241 |
| GLUL | 1 | 182354967 | 182354967 | C | T | exonic | synonymousSNV | 0.00003185 | rs200833075 |
| DHX9 | 1 | 182856330 | 182856344 | GGAGGCTATGGTAGC | - | exonic | nonframeshiftdeletion | 0.0009 | rs533935555 |
| ZNF281 | 1 | 200376608 | 200376608 | T | G | exonic | synonymousSNV | 0.0063 | rs74547203 |
| KIF21B | 1 | 200941792 | 200941792 | G | C | UTR3 | . | . | . |
| PRELP | 1 | 203452996 | 203452996 | G | A | exonic | synonymousSNV | 0.0036 | rs139420607 |
| PIK3C2B | 1 | 204411688 | 204411688 | C | G | exonic | nonsynonymousSNV | 0.0023 | rs61763421 |
| PIK3C2B | 1 | 204438956 | 204438956 | A | G | UTR5 | . | 0.0035 | rs61760619 |
| MDM4 | 1 | 204518457 | 204518457 | A | C | exonic | nonsynonymousSNV | 0.0032 | rs41299595 |
| IL20 | 1 | 207042033 | 207042033 | G | A | UTR3 | . | 0.0163 | rs2232363 |
| CD34 | 1 | 208060846 | 208060846 | G | A | UTR3 | . | 0.0053 | rs150401539 |
| NEK2 | 1 | 211848948 | 211848948 | G | T | UTR5 | . | 0.0047 | rs56231831 |
| KCTD3 | 1 | 215794924 | 215794924 | T | - | UTR3 | . | 0.0101 | rs370298874 |
| USH2A | 1 | 216040348 | 216040348 | C | T | splicing | . | . | . |
| ESRRG | 1 | 216680125 | 216680125 | A | G | UTR3 | . | 0.0017 | rs182373024 |
| HHIPL2 | 1 | 222721226 | 222721226 | G | T | exonic | nonsynonymousSNV | . | . |
| MIA3 | 1 | 222801661 | 222801661 | A | T | exonic | nonsynonymousSNV | 0.0089 | rs142088763 |
| CAPN2 | 1 | 223962537 | 223962537 | G | T | exonic | nonsynonymousSNV | 0.00003196 | . |
| WDR26 | 1 | 224588736 | 224588736 | A | G | exonic | synonymousSNV | 0.0025 | rs140546120 |
| DNAH14 | 1 | 225284923 | 225284923 | A | G | exonic | nonsynonymousSNV | . | . |
| DNAH14 | 1 | 225306982 | 225306982 | C | T | exonic | synonymousSNV | 0.0033 | rs150618371 |
| DNAH14 | 1 | 225334919 | 225334922 | AATT | - | exonic | frameshiftdeletion | 0.0025 | rs563040491 |
| DNAH14 | 1 | 225380564 | 225380564 | A | G | exonic | nonsynonymousSNV | 0.0058 | rs191528375 |
| DNAH14 | 1 | 225533910 | 225533910 | T | C | exonic | nonsynonymousSNV | 0.0005 | rs201079664 |
| LBR | 1 | 225611688 | 225611688 | G | A | exonic | synonymousSNV | 0.0071 | rs2230416 |
| SRP9 | 1 | 225977065 | 225977065 | G | A | UTR3 | . | . | rs1030783572 |
| TMEM63A | 1 | 226055624 | 226055624 | C | T | exonic | nonsynonymousSNV | 0.0029 | rs115600896 |
| PARP1 | 1 | 226579963 | 226579963 | A | G | exonic | synonymousSNV | 0.0021 | rs61750986 |
| PARP1 | 1 | 226579989 | 226579989 | T | C | exonic | nonsynonymousSNV | . | . |
| C1orf95 | 1 | 226789750 | 226789750 | G | A | UTR3 | . | . | rs772770695 |
| OBSCN | 1 | 228475975 | 228475975 | A | C | exonic | nonsynonymousSNV | . | . |
| NUP133 | 1 | 229586347 | 229586347 | A | G | exonic | synonymousSNV | 0.00003185 | rs374077818 |
| C1orf198 | 1 | 230974257 | 230974257 | - | T | UTR3 | . | 0.0092 | rs200535534 |
| C1orf198 | 1 | 230974262 | 230974262 | A | T | UTR3 | . | 0.0064 | . |
| KIAA1804 | 1 | 233490704 | 233490704 | G | A | exonic | nonsynonymousSNV | 0.0004 | rs35465006 |
| HEATR1 | 1 | 236749128 | 236749128 | A | C | exonic | nonsynonymousSNV | 0.0091 | rs41308194 |
| HEATR1 | 1 | 236758882 | 236758882 | T | C | exonic | nonsynonymousSNV | 0.0027 | rs144891600 |
| GRHL1 | 2 | 10141173 | 10141173 | C | T | UTR3 | . | 0.0112 | rs76304687 |
| ATP6V1C2 | 2 | 10904524 | 10904524 | C | T | exonic | synonymousSNV | 0.0013 | rs143556651 |
| ATP6V1C2 | 2 | 10924584 | 10924584 | C | A | UTR3 | . | 0.0015 | rs148675677 |
| ATP6V1C2 | 2 | 10924613 | 10924613 | C | T | UTR3 | . | 0.0047 | rs141845870 |
| SLC5A6 | 2 | 27422965 | 27422967 | CCA | - | UTR3 | . | 0.0049 | rs376605944 |
| ARHGEF33 | 2 | 39201095 | 39201095 | G | A | UTR3 | . | 0.0012 | rs191032578 |
| EPAS1 | 2 | 46611853 | 46611853 | C | T | UTR3 | . | 0.0031 | rs142847751 |
| XPO1 | 2 | 61719182 | 61719182 | A | C | exonic | synonymousSNV | 0.0008 | rs143005485 |
| MTHFD2 | 2 | 74425701 | 74425701 | C | T | UTR5 | . | 0.0027 | rs11687991 |
| DQX1 | 2 | 74750290 | 74750290 | C | T | exonic | nonsynonymousSNV | . | . |
| SEMA4F | 2 | 74900588 | 74900588 | G | C | exonic | synonymousSNV | 0.0044 | rs34617503 |
| EVA1A | 2 | 75719537 | 75719537 | - | TAA | UTR3 | . | 0.0743 | . |
| DNAH6 | 2 | 84831486 | 84831486 | G | A | exonic | synonymousSNV | 0.0017 | rs188063631 |
| PLGLB1,PLGLB2,RGPD1 | 2 | 88056462 | 88056466 | TTTCC | - | UTR3 | . | 0.0299 | rs199840058 |
| MAL | 2 | 95719316 | 95719316 | A | C | UTR3 | . | 0.0007 | rs1056989608 |
| ASTL | 2 | 96798356 | 96798356 | A | G | exonic | nonsynonymousSNV | 0.0008 | rs139152188 |
| TMEM131 | 2 | 98373635 | 98373635 | C | T | exonic | nonsynonymousSNV | 0.0007 | rs199651021 |
| RFX8 | 2 | 102013928 | 102013928 | G | A | UTR3 | . | 0.0167 | rs115333912 |
| RGPD3 | 2 | 107039737 | 107039737 | G | A | exonic | synonymousSNV | 0.0063 | rs200935720 |
| RGPD4 | 2 | 108475928 | 108475928 | C | T | exonic | nonsynonymousSNV | 0.0015 | rs143215949 |
| RGPD4 | 2 | 108507774 | 108507774 | A | G | UTR3 | . | 0.0474 | rs879387806 |
| RGPD4 | 2 | 108507777 | 108507777 | G | A | UTR3 | . | 0.0458 | . |
| RGPD4 | 2 | 108507996 | 108507996 | A | T | UTR3 | . | 0.0024 | . |
| GCC2 | 2 | 109124837 | 109124837 | A | T | UTR3 | . | 0.012 | rs539501211 |
| GCC2 | 2 | 109125778 | 109125778 | A | G | UTR3 | . | 0.0155 | rs149196384 |
| ZC3H6 | 2 | 113090619 | 113090619 | A | G | UTR3 | . | 0.0152 | rs147719341 |
| RGPD5,RGPD8 | 2 | 113145839 | 113145839 | G | A | exonic | synonymousSNV | 0.0138 | rs200960604 |
| SLC20A1 | 2 | 113404974 | 113404974 | A | C | exonic | synonymousSNV | 0.0063 | rs139798830 |
| CBWD2 | 2 | 114202557 | 114202557 | G | A | exonic | nonsynonymousSNV | . | . |
| FOXD4L1 | 2 | 114257266 | 114257266 | C | T | exonic | nonsynonymousSNV | 0.0031 | rs142136016 |
| CLASP1 | 2 | 122286263 | 122286263 | G | A | exonic | nonsynonymousSNV | 0.0009 | rs199865832 |
| MAP3K2 | 2 | 128087544 | 128087544 | T | C | exonic | nonsynonymousSNV | 0.0014 | rs56307783 |
| SFT2D3 | 2 | 128460866 | 128460866 | C | G | UTR3 | . | 0.0178 | rs143249179 |
| WDR33 | 2 | 128462192 | 128462192 | A | - | UTR3 | . | 0.0001 | rs982449691 |
| WDR33 | 2 | 128495265 | 128495265 | A | G | UTR3 | . | 0.0243 | rs11686607 |
| SMPD4 | 2 | 130930014 | 130930014 | G | A | exonic | synonymousSNV | . | . |
| WTH3DI | 2 | 132121610 | 132121610 | C | A | UTR5 | . | 0.0069 | rs537845454 |
| WTH3DI | 2 | 132121611 | 132121611 | A | G | UTR5 | . | 0.0065 | rs556157615 |
| WTH3DI | 2 | 132121626 | 132121626 | A | G | UTR5 | . | 0.0016 | . |
| WTH3DI | 2 | 132121629 | 132121629 | G | A | UTR5 | . | 0.0011 | . |
| RAB3GAP1 | 2 | 135926205 | 135926205 | C | G | exonic | nonsynonymousSNV | 0.0005 | rs77535003 |
| SPOPL | 2 | 139316900 | 139316900 | G | A | exonic | nonsynonymousSNV | 0.0034 | rs145753116 |
| RND3 | 2 | 151343986 | 151343986 | - | T | UTR5 | . | 0.069 | . |
| NMI | 2 | 152127169 | 152127169 | C | A | UTR3 | . | . | rs41268703 |
| RBMS1 | 2 | 161130855 | 161130855 | - | C | UTR3 | . | 0.0208 | rs557809075 |
| B3GALT1 | 2 | 168726975 | 168726975 | G | A | UTR3 | . | 0.0025 | rs150648556 |
| LRP2 | 2 | 170127559 | 170127559 | G | A | exonic | synonymousSNV | 0.0077 | rs141180155 |
| HOXD11 | 2 | 176973958 | 176973958 | T | A | UTR3 | . | . | . |
| HOXD10 | 2 | 176984426 | 176984426 | - | A | UTR3 | . | 0.0184 | rs113041382 |
| OSBPL6 | 2 | 179188974 | 179188974 | C | T | exonic | nonsynonymousSNV | 0.0013 | rs34874235 |
| PRKRA | 2 | 179315718 | 179315718 | C | T | exonic | nonsynonymousSNV | . | rs933550231 |
| TTN | 2 | 179435418 | 179435418 | T | C | exonic | synonymousSNV | 0.0037 | rs56151652 |
| TTN | 2 | 179517605 | 179517605 | G | A | exonic | nonsynonymousSNV | 0.0038 | rs150223722 |
| PDE1A | 2 | 183051216 | 183051216 | G | A | exonic | nonsynonymousSNV | . | rs553030991 |
| FSIP2 | 2 | 186657184 | 186657184 | T | C | exonic | nonsynonymousSNV | . | . |
| ASNSD1 | 2 | 190535254 | 190535254 | T | C | exonic | synonymousSNV | . | rs368203972 |
| SLC39A10 | 2 | 196600464 | 196600466 | TAT | - | UTR3 | . | . | rs936105036 |
| SLC39A10 | 2 | 196600532 | 196600532 | T | - | UTR3 | . | . | . |
| DNAH7 | 2 | 196681546 | 196681546 | G | A | exonic | synonymousSNV | 0.0073 | rs139963761 |
| ANKRD44 | 2 | 197854249 | 197854249 | T | C | UTR3 | . | 0.0111 | rs78756495 |
| AOX1 | 2 | 201521609 | 201521609 | C | T | exonic | synonymousSNV | 0.0033 | rs114924183 |
| BZW1 | 2 | 201688031 | 201688031 | C | T | UTR3 | . | 0.0007 | rs140367396 |
| CLK1 | 2 | 201722448 | 201722448 | A | C | exonic | synonymousSNV | 0.0003 | rs147232813 |
| ABI2 | 2 | 204292410 | 204292410 | C | T | UTR3 | . | 0.003 | rs141958154 |
| RAPH1 | 2 | 204299036 | 204299036 | C | T | UTR3 | . | 0.0006 | rs185298048 |
| NRP2 | 2 | 206592624 | 206592624 | C | T | exonic | nonsynonymousSNV | 0.0024 | rs114144673 |
| ZDBF2 | 2 | 207175023 | 207175023 | A | C | exonic | nonsynonymousSNV | 0.0075 | rs150375195 |
| DYTN | 2 | 207527981 | 207527981 | C | A | exonic | nonsynonymousSNV | 0.0038 | rs184887506 |
| CREB1 | 2 | 208462173 | 208462173 | T | G | UTR3 | . | 0.0018 | rs143587811 |
| MAP2 | 2 | 210558134 | 210558134 | G | T | exonic | nonsynonymousSNV | 0.0101 | rs41265969 |
| MAP2 | 2 | 210598605 | 210598605 | - | A | UTR3 | . | 0.0917 | rs137944482 |
| BARD1 | 2 | 215593618 | 215593618 | T | C | exonic | nonsynonymousSNV | 0.00006368 | rs149262370 |
| FN1 | 2 | 216284009 | 216284009 | C | T | exonic | nonsynonymousSNV | 0.0023 | rs147831535 |
| FN1 | 2 | 216295523 | 216295523 | C | T | exonic | synonymousSNV | . | rs772202402 |
| CATIP | 2 | 219227599 | 219227599 | G | A | exonic | nonsynonymousSNV | 0.0014 | rs138318485 |
| VIL1 | 2 | 219303399 | 219303399 | G | C | exonic | nonsynonymousSNV | 0.0004 | rs145234426 |
| PTPRN | 2 | 220154670 | 220154670 | G | T | UTR3 | . | 0.0159 | rs62191854 |
| DNPEP | 2 | 220250232 | 220250232 | G | C | exonic | nonsynonymousSNV | 0.0075 | rs115897075 |
| EPHA4 | 2 | 222437053 | 222437053 | T | C | UTR5 | . | 0.0003 | rs528282896 |
| PAX3 | 2 | 223066017 | 223066017 | - | CC | UTR3 | . | 0.0127 | rs199895781 |
| PAX3 | 2 | 223158385 | 223158385 | A | G | UTR3 | . | 0.015 | rs28945096 |
| GRM7 | 3 | 7620900 | 7620900 | A | G | exonic | synonymousSNV | . | rs567253699 |
| GRM7 | 3 | 7782551 | 7782551 | C | T | UTR3 | . | 0.0133 | rs17726576 |
| FANCD2 | 3 | 10143086 | 10143086 | - | A | UTR3 | . | 0.0203 | rs138111025 |
| SATB1 | 3 | 18390353 | 18390353 | - | A | UTR3 | . | 0.0148 | rs149083875 |
| RPL15 | 3 | 23961416 | 23961416 | G | C | UTR3 | . | 0.00003187 | . |
| LRRC3B | 3 | 26752064 | 26752064 | A | G | UTR3 | . | 0.0056 | rs73059389 |
| ZDHHC3 | 3 | 44968019 | 44968019 | - | T | UTR3 | . | 0.00006676 | rs34221393 |
| CCDC51 | 3 | 48475353 | 48475353 | G | A | UTR5 | . | 0.0165 | rs149306159 |
| IP6K2 | 3 | 48726170 | 48726170 | T | G | exonic | nonsynonymousSNV | 0.005 | rs150229636 |
| C3orf62 | 3 | 49308392 | 49308392 | A | - | UTR3 | . | 0.0011 | rs1041616473 |
| CISH | 3 | 50645296 | 50645296 | G | A | exonic | synonymousSNV | 0.0043 | rs61737505 |
| DNAH1 | 3 | 52384594 | 52384594 | A | G | exonic | nonsynonymousSNV | 0.0047 | rs61734644 |
| ERC2 | 3 | 55543453 | 55543453 | T | C | UTR3 | . | 0.00006372 | rs756839814 |
| IL17RD | 3 | 57125365 | 57125365 | A | G | UTR3 | . | 0.0039 | rs74560114 |
| FLNB | 3 | 58157850 | 58157850 | G | A | UTR3 | . | 0.0077 | rs115935959 |
| ACOX2 | 3 | 58490887 | 58490887 | A | G | UTR3 | . | 0.0083 | rs75857311 |
| PSMD6 | 3 | 63996607 | 63996607 | G | A | exonic | synonymousSNV | 0.0015 | rs34590097 |
| EBLN2 | 3 | 73111724 | 73111724 | T | G | exonic | stopgain | 0.0061 | rs2231925 |
| CNTN3 | 3 | 74414810 | 74414810 | G | T | exonic | synonymousSNV | . | . |
| CRYBG3 | 3 | 97594155 | 97594155 | G | C | exonic | nonsynonymousSNV | 0.0025 | rs187682797 |
| ADGRG7 | 3 | 100365491 | 100365491 | G | A | exonic | nonsynonymousSNV | 0.0013 | rs143826267 |
| FBXO40 | 3 | 121341019 | 121341019 | A | T | exonic | nonsynonymousSNV | 0.0052 | rs142436040 |
| HCLS1 | 3 | 121350650 | 121350650 | T | C | UTR3 | . | 0.0044 | rs111914785 |
| ITGB5 | 3 | 124578212 | 124578212 | C | G | exonic | nonsynonymousSNV | 0.0044 | rs112151655 |
| ZNF148 | 3 | 124949115 | 124949115 | C | T | UTR3 | . | 0.0043 | rs13323832 |
| MCM2 | 3 | 127327309 | 127327309 | G | A | exonic | nonsynonymousSNV | 0.0074 | rs3087450 |
| COL6A5 | 3 | 130107567 | 130107567 | T | G | exonic | nonsynonymousSNV | 0.0053 | rs144238271 |
| AMOTL2 | 3 | 134089758 | 134089758 | C | T | exonic | nonsynonymousSNV | 0.0086 | rs143351962 |
| CPA3 | 3 | 148601469 | 148601469 | C | T | exonic | nonsynonymousSNV | 0.0056 | rs140470073 |
| LEKR1 | 3 | 156547106 | 156547106 | A | G | UTR5 | . | 0.0032 | rs192904546 |
| SLC2A2 | 3 | 170732426 | 170732426 | G | A | exonic | nonsynonymousSNV | 0.0038 | rs7637863 |
| GHSR | 3 | 172162938 | 172162938 | G | A | UTR3 | . | 0.0002 | rs376078792 |
| NCEH1 | 3 | 172349912 | 172349912 | G | A | UTR3 | . | 0.0081 | rs187776749 |
| ZNF639 | 3 | 179051234 | 179051234 | A | G | exonic | nonsynonymousSNV | 0.0005 | rs139089340 |
| KLHL24 | 3 | 183397256 | 183397256 | - | A | UTR3 | . | 0.0003 | rs889354945 |
| YEATS2 | 3 | 183508690 | 183508690 | G | C | exonic | nonsynonymousSNV | 0.00006368 | rs553457288 |
| ATP13A3 | 3 | 194169223 | 194169223 | G | A | exonic | synonymousSNV | 0.001 | rs185170011 |
| ZFYVE28 | 4 | 2272295 | 2272295 | C | A | UTR3 | . | . | rs1002537321 |
| LYAR | 4 | 4269487 | 4269487 | - | TT | UTR3 | . | 0.1474 | rs10689966 |
| ACOX3 | 4 | 8401319 | 8401319 | G | A | exonic | synonymousSNV | 0.0027 | rs114063923 |
| PPARGC1A | 4 | 23796952 | 23796952 | A | T | UTR3 | . | 0.0304 | rs2932963 |
| SLAIN2 | 4 | 48343733 | 48343735 | GGC | - | UTR5 | . | 0 | rs745600491 |
| UBA6 | 4 | 68483772 | 68483774 | CAC | - | UTR3 | . | 0.0014 | . |
| SULT1E1 | 4 | 70707559 | 70707559 | - | A | UTR3 | . | . | . |
| EPGN | 4 | 75174838 | 75174838 | C | T | exonic | synonymousSNV | 0.0019 | rs148495973 |
| FAM47E,FAM47E-STBD1 | 4 | 77184996 | 77184996 | C | T | exonic | nonsynonymousSNV | 0.0112 | rs61730247 |
| FRAS1 | 4 | 79188491 | 79188491 | G | A | exonic | nonsynonymousSNV | 0.0008 | rs186811333 |
| FRAS1 | 4 | 79432453 | 79432453 | G | A | exonic | nonsynonymousSNV | 0.0053 | rs61729366 |
| PRDM8 | 4 | 81124893 | 81124893 | C | - | UTR3 | . | 0.0002 | . |
| HELQ | 4 | 84376794 | 84376794 | T | C | exonic | nonsynonymousSNV | 0.0049 | rs141700135 |
| FAM175A | 4 | 84384688 | 84384688 | C | T | exonic | nonsynonymousSNV | 0.0009 | rs114513239 |
| KLHL8 | 4 | 88116798 | 88116798 | C | T | UTR5 | . | 0.0219 | rs62306559 |
| HERC3 | 4 | 89601300 | 89601300 | T | C | exonic | synonymousSNV | 0.0032 | rs75022724 |
| TET2 | 4 | 106157003 | 106157003 | A | G | exonic | nonsynonymousSNV | . | rs962385281 |
| INTS12 | 4 | 106604070 | 106604070 | C | T | exonic | synonymousSNV | 0.0027 | rs145174807 |
| DKK2 | 4 | 107847073 | 107847073 | C | T | exonic | nonsynonymousSNV | 0.0001 | rs149779089 |
| COL25A1 | 4 | 109740435 | 109740435 | C | T | exonic | synonymousSNV | 0.00003186 | rs774443798 |
| ANK2 | 4 | 114294537 | 114294537 | G | A | exonic | nonsynonymousSNV | 0.0019 | rs45454496 |
| ARSJ | 4 | 114822971 | 114822971 | A | G | UTR3 | . | 0.0086 | rs77948545 |
| SYNPO2 | 4 | 119979243 | 119979243 | G | A | UTR3 | . | 0.1726 | rs62328678 |
| CCNA2,EXOSC9 | 4 | 122738137 | 122738137 | T | G | UTR3 | . | . | rs534409022 |
| DCHS2 | 4 | 155158284 | 155158284 | A | C | exonic | stopgain | 0.0018 | rs146298768 |
| DCHS2 | 4 | 155219467 | 155219467 | C | T | exonic | nonsynonymousSNV | 0.0005 | rs149550434 |
| DCHS2 | 4 | 155219540 | 155219540 | C | A | exonic | nonsynonymousSNV | 0.0018 | rs141467714 |
| ETFDH | 4 | 159593536 | 159593536 | A | C | UTR5 | . | . | rs545138897 |
| SH3RF1 | 4 | 170015927 | 170015927 | - | T | UTR3 | . | 0.0053 | . |
| BASP1 | 5 | 17276743 | 17276745 | AAA | - | UTR3 | . | . | rs878908182 |
| CDH6 | 5 | 31323058 | 31323058 | T | C | exonic | synonymousSNV | 0.0071 | rs146257998 |
| PDZD2 | 5 | 32074683 | 32074683 | G | A | exonic | synonymousSNV | 0.0034 | rs80096746 |
| RICTOR | 5 | 38940233 | 38940233 | A | - | UTR3 | . | 0.0008 | . |
| C9 | 5 | 39289043 | 39289043 | A | G | exonic | nonsynonymousSNV | 0.0016 | rs141645272 |
| C6 | 5 | 41261527 | 41261527 | C | T | UTR5 | . | 0.0106 | rs74627458 |
| HMGCS1 | 5 | 43290869 | 43290869 | T | - | UTR3 | . | 0.0097 | rs560924921 |
| MRPS30 | 5 | 44809433 | 44809433 | - | GAGCCC | exonic | nonframeshiftinsertion | 0.0037 | rs779308521 |
| GZMK | 5 | 54329605 | 54329605 | G | A | exonic | nonsynonymousSNV | 0.0029 | rs146429265 |
| SETD9 | 5 | 56212764 | 56212764 | A | G | UTR3 | . | 0.0062 | rs112920436 |
| MIER3 | 5 | 56217189 | 56217189 | A | C | UTR3 | . | 0.007 | rs111845852 |
| ACTBL2 | 5 | 56777007 | 56777007 | T | - | UTR3 | . | 0.0002 | . |
| JMY | 5 | 78532403 | 78532403 | C | A | UTR5 | . | 0.0031 | rs572119648 |
| SEPT8 | 5 | 132094410 | 132094410 | G | A | UTR3 | . | 0.0188 | rs114375386 |
| AFF4 | 5 | 132216256 | 132216256 | A | G | UTR3 | . | . | . |
| WDR55 | 5 | 140048355 | 140048355 | A | G | exonic | nonsynonymousSNV | . | . |
| PCDHB11 | 5 | 140580941 | 140580941 | G | A | exonic | nonsynonymousSNV | 0.0047 | rs138686663 |
| PCDHB11 | 5 | 140580967 | 140580967 | T | G | exonic | synonymousSNV | 0.0073 | rs149572646 |
| PCDHGA2 | 5 | 140720557 | 140720557 | C | T | exonic | synonymousSNV | 0.00006379 | rs770733671 |
| PCDHGA6 | 5 | 140754692 | 140754692 | G | A | exonic | nonsynonymousSNV | . | . |
| FAT2 | 5 | 150887120 | 150887120 | C | T | exonic | nonsynonymousSNV | 0.0012 | rs138750878 |
| GRIA1 | 5 | 153190599 | 153190599 | C | T | exonic | synonymousSNV | . | . |
| MFAP3 | 5 | 153434893 | 153434893 | T | C | UTR3 | . | 0.0041 | rs147223582 |
| MAT2B | 5 | 162930287 | 162930287 | T | G | UTR5 | . | 0.018 | rs75230624 |
| DSP | 6 | 7583532 | 7583532 | C | T | exonic | nonsynonymousSNV | . | rs756529985 |
| SLC35B3 | 6 | 8434635 | 8434636 | AT | - | UTR5 | . | 0.0003 | rs745467152 |
| SLC35B3 | 6 | 8434637 | 8434637 | T | C | UTR5 | . | 0.0003 | rs746943395 |
| TBC1D7 | 6 | 13316909 | 13316909 | G | T | exonic | nonsynonymousSNV | 0.0024 | rs80189640 |
| ATXN1 | 6 | 16303048 | 16303048 | G | T | UTR3 | . | 0.0053 | rs45496399 |
| ID4 | 6 | 19840259 | 19840259 | A | G | UTR3 | . | 0.00003186 | rs753758490 |
| LRRC16A | 6 | 25279841 | 25279841 | T | - | UTR5 | . | . | . |
| HIST1H2BA | 6 | 25727471 | 25727471 | C | G | exonic | nonsynonymousSNV | . | rs767245397 |
| HIST1H1E | 6 | 26156953 | 26156953 | C | T | exonic | nonsynonymousSNV | 0.0006 | rs201133864 |
| BTN3A2 | 6 | 26377069 | 26377069 | C | T | UTR3 | . | 0.0157 | rs111568057 |
| BTN3A3 | 6 | 26452211 | 26452211 | C | T | exonic | nonsynonymousSNV | 0.0062 | rs148819206 |
| ZKSCAN3 | 6 | 28336241 | 28336241 | - | T | UTR3 | . | 0.0003 | rs561542874 |
| GPX6 | 6 | 28473531 | 28473531 | C | G | exonic | unknown | 0.0055 | rs35394555 |
| OR2B3 | 6 | 29054923 | 29054923 | A | G | exonic | nonsynonymousSNV | 0.0079 | rs149723131 |
| OR5V1 | 6 | 29323245 | 29323245 | G | C | exonic | nonsynonymousSNV | 0.0071 | rs41271546 |
| MOG | 6 | 29627313 | 29627313 | A | G | exonic | synonymousSNV | 0.008 | rs34758289 |
| LOC554223 | 6 | 29765055 | 29765055 | T | C | UTR3 | . | 0.0355 | rs75904618 |
| LOC554223 | 6 | 29765061 | 29765061 | T | C | UTR3 | . | 0.031 | rs75220593 |
| PPP1R11 | 6 | 30038020 | 30038020 | C | T | UTR3 | . | 0.0007 | rs554336785 |
| RNF39 | 6 | 30038347 | 30038347 | C | - | UTR3 | . | 0.1061 | rs201540070 |
| MUC21 | 6 | 30954714 | 30954714 | A | T | exonic | synonymousSNV | 0.0061 | rs41288658 |
| MUC21 | 6 | 30954919 | 30954919 | A | G | exonic | nonsynonymousSNV | 0.0392 | rs41288679 |
| MUC21 | 6 | 30954920 | 30954920 | C | T | exonic | nonsynonymousSNV | 0.0298 | rs144503906 |
| MUC21 | 6 | 30954921 | 30954921 | C | G | exonic | synonymousSNV | 0.0297 | rs548248760 |
| HLA-B | 6 | 31321866 | 31321866 | C | T | UTR3 | . | 0.0276 | . |
| HLA-B | 6 | 31321867 | 31321867 | T | C | UTR3 | . | 0.0241 | . |
| C6orf47 | 6 | 31628397 | 31628397 | T | A | UTR5 | . | 0.0068 | rs148844907 |
| MSH5 | 6 | 31727989 | 31727989 | A | G | exonic | nonsynonymousSNV | 0.0064 | rs61748589 |
| C6orf48 | 6 | 31802721 | 31802721 | C | T | UTR5 | . | 0.0056 | rs17207608 |
| DXO | 6 | 31940029 | 31940029 | G | A | UTR5 | . | 0.0047 | rs75831035 |
| CYP21A2 | 6 | 32008783 | 32008783 | C | T | exonic | nonsynonymousSNV | 0.0042 | rs6445 |
| TNXB | 6 | 32021414 | 32021414 | C | G | exonic | nonsynonymousSNV | 0.0000319 | rs61745355 |
| TNXB | 6 | 32023778 | 32023778 | T | A | exonic | nonsynonymousSNV | 0.00003188 | rs572852092 |
| TNXB | 6 | 32025841 | 32025841 | C | T | exonic | nonsynonymousSNV | . | rs757463918 |
| PBX2 | 6 | 32153420 | 32153422 | AAC | - | UTR3 | . | 0.0666 | rs201396941 |
| C6orf10 | 6 | 32339554 | 32339554 | C | T | UTR5 | . | 0.0038 | rs140147638 |
| HLA-DRB5 | 6 | 32487394 | 32487394 | G | A | exonic | synonymousSNV | 0.0022 | rs144981689 |
| HLA-DRB5 | 6 | 32489873 | 32489873 | - | AG | exonic | frameshiftinsertion | 0.0088 | rs201466163 |
| HLA-DRB1 | 6 | 32546805 | 32546805 | - | A | UTR3 | . | 0.02 | rs764735614 |
| HLA-DRB1 | 6 | 32552017 | 32552017 | G | C | exonic | nonsynonymousSNV | 0.0885 | rs1059582 |
| HLA-DQB1 | 6 | 32632627 | 32632627 | C | G | exonic | synonymousSNV | 0.012 | rs1130389 |
| HLA-DQB1 | 6 | 32632645 | 32632645 | G | T | exonic | synonymousSNV | 0.0049 | rs9274386 |
| HLA-DQB1 | 6 | 32632646 | 32632646 | G | T | exonic | nonsynonymousSNV | 0.0054 | rs9274387 |
| HLA-DQA2 | 6 | 32712967 | 32712967 | C | G | exonic | nonsynonymousSNV | 0.00003288 | rs758244642 |
| HLA-DQA2 | 6 | 32712977 | 32712977 | C | T | exonic | nonsynonymousSNV | 0.0004 | rs200745258 |
| HLA-DQA2 | 6 | 32712979 | 32712979 | C | T | exonic | synonymousSNV | 0.0005 | rs202183183 |
| PSMB8 | 6 | 32809965 | 32809965 | C | T | exonic | synonymousSNV | 0.0034 | rs41270492 |
| TAP1 | 6 | 32816448 | 32816448 | G | A | exonic | nonsynonymousSNV | 0.0024 | rs2228106 |
| TAPBP | 6 | 33281504 | 33281504 | C | A | exonic | nonsynonymousSNV | 0.0038 | rs45583737 |
| TAPBP | 6 | 33281505 | 33281505 | C | A | exonic | synonymousSNV | 0.0038 | rs45501592 |
| KIFC1 | 6 | 33373341 | 33373341 | C | T | exonic | nonsynonymousSNV | 0.006 | rs7770412 |
| PACSIN1 | 6 | 34499387 | 34499387 | T | C | exonic | nonsynonymousSNV | 0.0057 | rs149949488 |
| PPARD | 6 | 35391922 | 35391922 | G | A | exonic | synonymousSNV | 0.0016 | rs138479838 |
| PNPLA1 | 6 | 36262125 | 36262125 | C | T | exonic | synonymousSNV | . | . |
| CMTR1 | 6 | 37448763 | 37448763 | G | A | UTR3 | . | 0.0065 | rs112485104 |
| MDGA1 | 6 | 37619853 | 37619853 | T | C | exonic | nonsynonymousSNV | 0.0005 | rs112648384 |
| MDGA1 | 6 | 37664706 | 37664706 | A | C | UTR5 | . | 0.0057 | rs113631515 |
| NCR2 | 6 | 41309618 | 41309618 | G | A | exonic | nonsynonymousSNV | 0.0101 | rs34456049 |
| FRS3 | 6 | 41738159 | 41738159 | C | T | UTR3 | . | 0.0117 | rs192318400 |
| PEX6 | 6 | 42931861 | 42931861 | G | A | UTR3 | . | 0.0232 | rs1051218 |
| POLR1C | 6 | 43487122 | 43487122 | A | G | exonic | nonsynonymousSNV | 0.0014 | rs141471029 |
| NFKBIE | 6 | 44233403 | 44233403 | G | A | exonic | nonsynonymousSNV | 0.0104 | rs2233430 |
| RCAN2 | 6 | 46189511 | 46189511 | G | A | UTR3 | . | 0.0046 | rs1057102 |
| TDRD6 | 6 | 46669724 | 46669724 | A | G | UTR3 | . | 0.0045 | rs149494397 |
| EFHC1 | 6 | 52288483 | 52288483 | A | T | splicing | . | 0.1961 | rs901570801 |
| EFHC1 | 6 | 52288484 | 52288484 | G | T | splicing | . | 0.1569 | rs998616603 |
| EFHC1 | 6 | 52288585 | 52288585 | C | T | UTR5 | . | 0.0078 | rs192503387 |
| LGSN | 6 | 63989792 | 63989792 | A | - | UTR3 | . | 0.0002 | rs1019568155 |
| EYS | 6 | 66053934 | 66053934 | T | G | exonic | nonsynonymousSNV | 0.0063 | rs61753611 |
| RIMS1 | 6 | 73110554 | 73110554 | G | T | UTR3 | . | 0.0089 | rs55642181 |
| RIMS1 | 6 | 73110712 | 73110713 | CA | - | UTR3 | . | 0.00009811 | rs143889624 |
| SLC17A5 | 6 | 74304363 | 74304363 | G | C | UTR3 | . | . | rs1009337696 |
| HMGN3 | 6 | 79911546 | 79911546 | - | T | UTR3 | . | 0.0017 | . |
| C6orf165 | 6 | 88123596 | 88123596 | C | T | exonic | synonymousSNV | 0.0061 | rs117489265 |
| MDN1 | 6 | 90385909 | 90385909 | G | A | exonic | nonsynonymousSNV | 0.0001 | rs116630523 |
| FUT9 | 6 | 96652235 | 96652235 | T | C | UTR3 | . | 0.0087 | rs139604379 |
| GRIK2 | 6 | 102483356 | 102483356 | C | T | exonic | synonymousSNV | 0.000032 | rs3213607 |
| PREP | 6 | 105725779 | 105725779 | A | - | UTR3 | . | 0.0124 | rs571897770 |
| WASF1 | 6 | 110499804 | 110499804 | G | - | UTR5 | . | 0.0285 | rs144619863 |
| WASF1 | 6 | 110499805 | 110499805 | T | A | UTR5 | . | 0.0286 | rs115103398 |
| SASH1 | 6 | 148865771 | 148865771 | A | G | exonic | synonymousSNV | 0.0024 | rs61996300 |
| UST | 6 | 149396925 | 149396925 | C | T | UTR3 | . | 0.0007 | rs187743603 |
| SYNE1 | 6 | 152784621 | 152784621 | T | C | exonic | nonsynonymousSNV | 0.0068 | rs9397509 |
| OPRM1 | 6 | 154411110 | 154411110 | C | G | exonic | nonsynonymousSNV | 0.0036 | rs17174794 |
| TMEM242 | 6 | 157743741 | 157743741 | T | C | exonic | nonsynonymousSNV | 0.0077 | rs187769654 |
| SNX9 | 6 | 158349699 | 158349699 | C | T | exonic | nonsynonymousSNV | 0.0057 | rs151179291 |
| IGF2R | 6 | 160484580 | 160484580 | C | T | exonic | synonymousSNV | 0.0011 | rs143709688 |
| PARK2 | 6 | 161771036 | 161771037 | GT | - | UTR3 | . | 0.0000646 | rs949516613 |
| QKI | 6 | 163987170 | 163987170 | G | A | UTR3 | . | 0.0066 | rs139890146 |
| TCP10L2 | 6 | 167594169 | 167594169 | G | A | exonic | nonsynonymousSNV | 0.0049 | rs201976311 |
| ZMIZ2 | 7 | 44806089 | 44806089 | G | A | exonic | nonsynonymousSNV | 0.01 | rs71548246 |
| NCF1 | 7 | 74193665 | 74193665 | T | G | exonic | nonsynonymousSNV | 0.0008 | rs144018361 |
| NCF1 | 7 | 74202933 | 74202933 | C | T | exonic | synonymousSNV | 0.0014 | rs587594457 |
| GTF2IRD2 | 7 | 74211705 | 74211705 | G | A | exonic | nonsynonymousSNV | 0.0076 | rs12375120 |
| ABCB4 | 7 | 87074281 | 87074281 | - | A | exonic | frameshiftinsertion | 0.00003477 | rs765735641 |
| ADAM22 | 7 | 87825858 | 87825858 | A | G | UTR3 | . | 0.0009 | rs377744635 |
| CDK14 | 7 | 90225830 | 90225830 | G | A | UTR5 | . | 0.0227 | rs11760706 |
| PEG10 | 7 | 94295454 | 94295454 | C | T | UTR3 | . | 0.006 | rs146181651 |
| TAF6 | 7 | 99705740 | 99705740 | G | C | exonic | nonsynonymousSNV | 0.00006382 | rs965767102 |
| MOSPD3 | 7 | 100211254 | 100211254 | G | C | exonic | nonsynonymousSNV | 0.0007 | rs137921044 |
| TFR2 | 7 | 100228655 | 100228655 | G | T | exonic | nonsynonymousSNV | 0.0028 | rs41303495 |
| ZAN | 7 | 100377288 | 100377288 | C | T | exonic | unknown | 0.0009 | rs368183208 |
| MUC12 | 7 | 100643259 | 100643259 | C | T | exonic | stopgain | 0.0005 | rs764167828 |
| AP1S1 | 7 | 100799988 | 100799988 | C | G | exonic | synonymousSNV | 0.0029 | rs148305541 |
| AP1S1 | 7 | 100804017 | 100804017 | C | T | UTR3 | . | 0.0034 | rs371696190 |
| MOGAT3 | 7 | 100839291 | 100839291 | T | A | exonic | nonsynonymousSNV | 0.0029 | rs139545520 |
| PLOD3 | 7 | 100860054 | 100860054 | C | T | exonic | synonymousSNV | 0.0032 | rs112321807 |
| CUX1 | 7 | 101893332 | 101893332 | G | A | UTR3 | . | 0.0105 | . |
| DOCK4 | 7 | 111846342 | 111846342 | G | A | UTR5 | . | 0.0242 | rs138530312 |
| TMEM168 | 7 | 112406409 | 112406409 | T | C | UTR3 | . | 0.0082 | rs139692654 |
| GPR85 | 7 | 112723448 | 112723448 | C | T | UTR3 | . | 0.0105 | rs56320353 |
| HILPDA | 7 | 128097963 | 128097963 | - | A | UTR3 | . | 0.01 | rs376818816 |
| FLNC | 7 | 128499294 | 128499294 | G | A | UTR3 | . | 0.0285 | rs117394163 |
| C7orf49 | 7 | 134853630 | 134853630 | T | C | exonic | synonymousSNV | . | . |
| MTRNR2L6 | 7 | 142374845 | 142374845 | C | T | UTR5 | . | 0.0092 | rs73166797 |
| EPHB6 | 7 | 142561747 | 142561747 | C | T | exonic | synonymousSNV | 0.0064 | rs56087487 |
| ANGPT2 | 8 | 6357207 | 6357207 | A | G | UTR3 | . | 0.0152 | rs2442474 |
| XKR5 | 8 | 6681244 | 6681244 | T | C | exonic | nonsynonymousSNV | 0.00003188 | rs753681366 |
| SPAG11B | 8 | 7320233 | 7320233 | G | A | exonic | synonymousSNV | 0.0019 | rs138525973 |
| SPAG11A,SPAG11B | 8 | 7706361 | 7706361 | C | T | exonic | synonymousSNV | 0.0021 | rs767505896 |
| SGK223 | 8 | 8234904 | 8234904 | C | T | exonic | nonsynonymousSNV | 0.0049 | rs201521501 |
| TNKS | 8 | 9627685 | 9627685 | C | T | exonic | synonymousSNV | 0.0005 | rs370028506 |
| TNKS | 8 | 9634341 | 9634341 | C | G | UTR3 | . | . | . |
| TNKS | 8 | 9639850 | 9639850 | T | A | UTR3 | . | 0.0004 | rs567402909 |
| PRSS55 | 8 | 10390537 | 10390537 | T | C | exonic | synonymousSNV | 0.0001 | rs182279550 |
| PRSS55 | 8 | 10411509 | 10411509 | G | A | exonic | synonymousSNV | 0.0003 | rs568815105 |
| XKR6 | 8 | 10755310 | 10755310 | T | - | UTR3 | . | 0.0005 | rs961222705 |
| USP17L7 | 8 | 11991241 | 11991241 | A | G | exonic | nonsynonymousSNV | 0.001 | rs754296160 |
| USP17L7 | 8 | 11991247 | 11991247 | A | T | exonic | nonsynonymousSNV | 0.0009 | rs752930744 |
| USP17L7 | 8 | 11991249 | 11991249 | A | G | exonic | synonymousSNV | 0.0009 | rs758637147 |
| USP17L7 | 8 | 11991253 | 11991253 | A | C | exonic | nonsynonymousSNV | 0.0009 | rs781325442 |
| USP17L2 | 8 | 11995066 | 11995066 | G | A | exonic | stopgain | 0.0008 | rs199744133 |
| FAM86B1 | 8 | 12039801 | 12039801 | G | T | UTR3 | . | 0.0648 | . |
| FAM86B1 | 8 | 12040290 | 12040290 | C | G | UTR3 | . | 0.0101 | rs376819154 |
| FAM86B1 | 8 | 12040822 | 12040822 | A | G | UTR3 | . | 0.0434 | rs752416360 |
| KIAA1456 | 8 | 12879538 | 12879538 | G | A | exonic | synonymousSNV | 0.0003 | rs755973996 |
| ZDHHC2 | 8 | 17079489 | 17079489 | G | T | UTR3 | . | 0.0125 | rs200415282 |
| R3HCC1 | 8 | 23152310 | 23152310 | G | A | exonic | synonymousSNV | 0.006 | rs117222031 |
| STC1 | 8 | 23700814 | 23700814 | - | T | UTR3 | . | 0.0003 | . |
| ESCO2 | 8 | 27645419 | 27645419 | A | C | exonic | nonsynonymousSNV | 0.0007 | rs143346057 |
| TOX | 8 | 59717983 | 59717983 | T | C | UTR3 | . | 0.0263 | rs62505114 |
| YTHDF3 | 8 | 64122594 | 64122594 | A | - | UTR3 | . | 0.0075 | rs200482681 |
| ARFGEF1 | 8 | 68130265 | 68130265 | C | A | exonic | nonsynonymousSNV | 0.0065 | rs142342517 |
| PREX2 | 8 | 69143714 | 69143715 | TT | - | UTR3 | . | . | . |
| TRPA1 | 8 | 72981337 | 72981337 | G | A | exonic | nonsynonymousSNV | 0.0008 | rs61758122 |
| UBE2W | 8 | 74704460 | 74704460 | A | G | UTR3 | . | 0.003 | rs574846023 |
| PI15 | 8 | 75761460 | 75761460 | C | A | exonic | nonsynonymousSNV | 0.00003187 | rs200826695 |
| CRISPLD1 | 8 | 75944618 | 75944621 | AAAC | - | UTR3 | . | 0.0125 | rs200279018 |
| CRISPLD1 | 8 | 75945637 | 75945637 | G | A | UTR3 | . | 0.0127 | rs138315327 |
| ZBTB10 | 8 | 81432215 | 81432218 | ATTA | - | UTR3 | . | 0.0056 | rs201350779 |
| MMP16 | 8 | 89339657 | 89339657 | C | G | UTR5 | . | . | . |
| SLC26A7 | 8 | 92374629 | 92374629 | G | A | exonic | nonsynonymousSNV | 0.0061 | rs145779342 |
| GDF6 | 8 | 97155120 | 97155120 | G | A | UTR3 | . | 0.0056 | rs191538930 |
| RIMS2 | 8 | 105264090 | 105264090 | - | A | UTR3 | . | . | . |
| ZHX2 | 8 | 123986493 | 123986493 | T | C | UTR3 | . | . | . |
| ZHX1 | 8 | 124262078 | 124262078 | T | - | UTR3 | . | 0.0218 | . |
| TG | 8 | 133984064 | 133984064 | G | A | exonic | nonsynonymousSNV | 0.0001 | rs199923962 |
| ZFAT | 8 | 135545200 | 135545200 | G | A | exonic | nonsynonymousSNV | . | . |
| FAM135B | 8 | 139164185 | 139164185 | T | C | exonic | nonsynonymousSNV | 0.0048 | rs114885489 |
| COL22A1 | 8 | 139706780 | 139706780 | A | G | exonic | synonymousSNV | . | rs771685487 |
| SLC24A2 | 9 | 19576929 | 19576929 | G | A | exonic | synonymousSNV | 0.0086 | rs112508827 |
| MLLT3 | 9 | 20346442 | 20346442 | C | T | exonic | synonymousSNV | . | . |
| FOCAD | 9 | 20944629 | 20944629 | G | A | exonic | synonymousSNV | 0.0063 | rs56037765 |
| KLHL9 | 9 | 21335322 | 21335333 | TGGGCCTGGGCT | - | UTR5 | . | 0.0246 | rs201092918 |
| TMEM215 | 9 | 32788927 | 32788927 | A | T | UTR3 | . | 0.0012 | rs62541977 |
| PRSS3 | 9 | 33796660 | 33796660 | T | C | exonic | synonymousSNV | 0.0002 | rs760851289 |
| CNTFR | 9 | 34551446 | 34551446 | G | A | UTR3 | . | 0.0111 | rs112702512 |
| ANKRD18A | 9 | 38615634 | 38615634 | T | C | exonic | nonsynonymousSNV | 0.0056 | rs187518165 |
| ZNF658 | 9 | 40774660 | 40774660 | A | G | exonic | synonymousSNV | 0.0016 | rs117327335 |
| ANKRD20A4 | 9 | 69391171 | 69391171 | G | A | exonic | nonsynonymousSNV | 0.0294 | rs200259990 |
| ANKRD20A4 | 9 | 69423983 | 69423983 | T | C | exonic | nonsynonymousSNV | 0.0104 | rs757181041 |
| ANKRD20A4 | 9 | 69424061 | 69424061 | T | C | exonic | nonsynonymousSNV | 0.0036 | rs748660775 |
| TMEM2 | 9 | 74298465 | 74298465 | - | TCAA | UTR3 | . | 0.0383 | . |
| C9orf47 | 9 | 91610636 | 91610636 | G | A | UTR3 | . | 0.0032 | rs183063895 |
| ROR2 | 9 | 94486310 | 94486310 | G | A | exonic | synonymousSNV | 0.0002 | rs146432734 |
| C9orf43 | 9 | 116187648 | 116187648 | - | GCA | exonic | nonframeshiftinsertion | . | . |
| CDK5RAP2 | 9 | 123287277 | 123287277 | G | A | exonic | nonsynonymousSNV | 0.0007 | rs145165171 |
| TRUB2 | 9 | 131076155 | 131076155 | C | T | exonic | nonsynonymousSNV | 0.0081 | rs41306706 |
| ODF2 | 9 | 131262512 | 131262512 | G | A | exonic | nonsynonymousSNV | 0.0048 | rs142129915 |
| LRRC8A | 9 | 131678626 | 131678626 | G | T | exonic | nonsynonymousSNV | . | . |
| LRRC8A | 9 | 131679225 | 131679225 | C | T | UTR3 | . | . | . |
| TOR1B | 9 | 132572629 | 132572629 | T | - | UTR3 | . | 0.0002 | . |
| USP20 | 9 | 132630457 | 132630457 | G | A | exonic | synonymousSNV | 0.002 | rs61760212 |
| PRDM12 | 9 | 133558185 | 133558185 | A | G | UTR3 | . | 0.00003184 | rs996092103 |
| ABL1 | 9 | 133759378 | 133759378 | C | T | exonic | synonymousSNV | 0.0023 | rs34372796 |
| NTNG2 | 9 | 135073541 | 135073541 | C | T | exonic | synonymousSNV | . | . |
| NMT2 | 10 | 15172179 | 15172179 | G | A | exonic | synonymousSNV | 0.0002 | rs139770446 |
| ITGA8 | 10 | 15655668 | 15655668 | G | A | exonic | nonsynonymousSNV | 0.00003184 | rs746556051 |
| C1QL3 | 10 | 16563566 | 16563566 | - | CCT | UTR5 | . | 0.0255 | rs548736528 |
| NEBL | 10 | 21071812 | 21071812 | A | G | UTR3 | . | 0.0066 | rs41277360 |
| C10orf113 | 10 | 21435317 | 21435317 | A | G | exonic | nonsynonymousSNV | 0.0007 | rs143569091 |
| BMI1,COMMD3-BMI1 | 10 | 22619256 | 22619256 | T | C | UTR3 | . | 0.0228 | rs113218050 |
| SPAG6 | 10 | 22676841 | 22676841 | A | T | exonic | synonymousSNV | . | rs370334559 |
| OTUD1 | 10 | 23730191 | 23730191 | C | G | UTR3 | . | 0.0078 | rs4586049 |
| ARHGAP21 | 10 | 24873881 | 24873881 | G | A | exonic | synonymousSNV | . | rs147583234 |
| MASTL | 10 | 27459716 | 27459716 | G | A | exonic | nonsynonymousSNV | 0.0097 | rs35571315 |
| AGAP4 | 10 | 46321880 | 46321880 | C | T | exonic | nonsynonymousSNV | 0.0652 | rs201179318 |
| BICC1 | 10 | 60588885 | 60588885 | A | C | UTR3 | . | 0.011 | rs75979288 |
| FAM13C,PHYHIPL | 10 | 61006956 | 61006956 | C | T | UTR3 | . | 0.0187 | rs78974041 |
| ARID5B | 10 | 63856070 | 63856070 | T | C | UTR3 | . | 0.0198 | rs140702604 |
| RTKN2 | 10 | 63957654 | 63957654 | C | T | UTR3 | . | 0.0013 | rs181762533 |
| ZNF365 | 10 | 64239835 | 64239835 | C | T | UTR3 | . | 0.0088 | rs144963168 |
| JMJD1C | 10 | 64927284 | 64927290 | TATACTT | - | UTR3 | . | 0.0155 | rs201673150 |
| JMJD1C | 10 | 64973978 | 64973978 | G | A | exonic | nonsynonymousSNV | 0.0087 | rs41274068 |
| REEP3 | 10 | 65281118 | 65281127 | CGGGCGGGGG | - | UTR5 | . | 0.0172 | rs773662275 |
| KIAA1279 | 10 | 70760271 | 70760271 | T | C | exonic | nonsynonymousSNV | 0.006 | rs62625033 |
| COL13A1 | 10 | 71648077 | 71648077 | C | A | exonic | nonsynonymousSNV | 0.0008 | rs202115626 |
| PPA1 | 10 | 71962714 | 71962714 | A | - | UTR3 | . | 0.0024 | rs537854671 |
| PALD1 | 10 | 72326567 | 72326567 | G | A | UTR3 | . | 0.0033 | rs142349683 |
| PRF1 | 10 | 72357635 | 72357635 | A | G | UTR3 | . | 0.0108 | rs74513535 |
| KAT6B | 10 | 76788660 | 76788668 | GAAGAGGAA | - | exonic | nonframeshiftdeletion | 0.0104 | rs544824146 |
| POLR3A | 10 | 79737146 | 79737146 | C | T | UTR3 | . | 0.0056 | rs146055367 |
| POLR3A | 10 | 79769647 | 79769647 | C | A | exonic | nonsynonymousSNV | 0.0087 | rs34588967 |
| ZMIZ1 | 10 | 81075270 | 81075270 | C | T | UTR3 | . | 0.0039 | rs45524835 |
| ZMIZ1 | 10 | 81076204 | 81076204 | A | G | UTR3 | . | . | rs527815323 |
| EIF5AL1 | 10 | 81275332 | 81275332 | C | T | UTR3 | . | 0.0236 | rs187635832 |
| ANXA11 | 10 | 81932595 | 81932595 | G | A | exonic | nonsynonymousSNV | 0.0045 | rs147334030 |
| WAPAL | 10 | 88196201 | 88196203 | ATG | - | UTR3 | . | 0.0011 | rs750456270 |
| ANKRD22 | 10 | 90591738 | 90591738 | G | A | exonic | nonsynonymousSNV | 0.0008 | rs117613865 |
| STAMBPL1 | 10 | 90673187 | 90673187 | G | A | exonic | synonymousSNV | 0.0058 | rs17850686 |
| EXOC6 | 10 | 94757245 | 94757245 | G | A | exonic | synonymousSNV | 0.0044 | rs34159839 |
| ARL3 | 10 | 104435594 | 104435594 | - | TA | UTR3 | . | 0.0162 | rs146155890 |
| CALHM2 | 10 | 105207301 | 105207301 | C | T | exonic | nonsynonymousSNV | 0.0043 | rs2232662 |
| DCLRE1A | 10 | 115602192 | 115602192 | T | A | exonic | nonsynonymousSNV | 0.0031 | rs11196530 |
| ADRB1 | 10 | 115805544 | 115805544 | C | T | UTR3 | . | 0.0022 | rs756675746 |
| AFAP1L2 | 10 | 116057027 | 116057027 | C | T | exonic | synonymousSNV | 0.006 | rs139572626 |
| KIAA1598 | 10 | 118645669 | 118645670 | TG | - | UTR3 | . | 0.0103 | rs141084580 |
| TACC2 | 10 | 123846421 | 123846421 | G | A | exonic | nonsynonymousSNV | 0.0014 | rs142508761 |
| OR51E2 | 11 | 4703762 | 4703762 | G | A | exonic | synonymousSNV | 0.0016 | rs12576387 |
| OR51F1 | 11 | 4791053 | 4791053 | G | C | exonic | nonsynonymousSNV | 0.0016 | rs148685376 |
| OVCH2 | 11 | 7723839 | 7723839 | A | C | exonic | nonsynonymousSNV | 0.00009557 | rs375950148 |
| C11orf16 | 11 | 8947437 | 8947437 | C | T | exonic | synonymousSNV | 0.00006372 | rs779180873 |
| FAR1 | 11 | 13750621 | 13750621 | G | A | UTR3 | . | 0.0121 | rs72866530 |
| C11orf58 | 11 | 16777048 | 16777048 | G | A | UTR3 | . | 0.0042 | rs117986410 |
| CSRP3 | 11 | 19209751 | 19209751 | G | A | exonic | synonymousSNV | 0.0052 | rs45476991 |
| MUC15 | 11 | 26582207 | 26582207 | C | T | UTR3 | . | 0.0103 | rs142185234 |
| EIF3M | 11 | 32605365 | 32605365 | C | G | UTR5 | . | 0.0032 | rs183375892 |
| API5 | 11 | 43364745 | 43364746 | TG | - | UTR3 | . | 0.0004 | rs776073125 |
| API5 | 11 | 43365154 | 43365154 | G | A | UTR3 | . | 0.0026 | rs534076943 |
| MAPK8IP1 | 11 | 45927353 | 45927353 | T | C | UTR3 | . | 0.005 | rs564463098 |
| AMBRA1 | 11 | 46564695 | 46564695 | C | A | exonic | nonsynonymousSNV | . | . |
| DDB2 | 11 | 47260551 | 47260551 | C | A | UTR3 | . | 0.0163 | rs4647760 |
| CELF1 | 11 | 47490447 | 47490447 | - | A | UTR3 | . | 0.0002 | rs146701169 |
| C11orf84 | 11 | 63594700 | 63594700 | G | A | UTR3 | . | 0.0056 | rs113443667 |
| COX8A | 11 | 63742263 | 63742263 | C | A | exonic | synonymousSNV | 0.0051 | rs61759492 |
| DNAJC4 | 11 | 64001633 | 64001633 | G | A | exonic | nonsynonymousSNV | 0.0034 | rs61734554 |
| PRDX5 | 11 | 64087269 | 64087269 | G | A | exonic | nonsynonymousSNV | 0.0036 | rs77269065 |
| GRM5 | 11 | 88300817 | 88300817 | C | T | exonic | synonymousSNV | 0.0052 | rs55933882 |
| FAT3 | 11 | 92086472 | 92086472 | A | C | exonic | synonymousSNV | 0.0004 | rs139731877 |
| MMP27 | 11 | 102565820 | 102565820 | C | A | exonic | nonsynonymousSNV | 0.0051 | rs35616217 |
| ATM | 11 | 108098576 | 108098576 | C | G | exonic | nonsynonymousSNV | 0.0076 | rs1800054 |
| COLCA1 | 11 | 111168821 | 111168821 | T | C | UTR5 | . | 0.0033 | rs139870543 |
| SIDT2 | 11 | 117052799 | 117052799 | A | G | exonic | nonsynonymousSNV | 0.0017 | rs145158897 |
| TMPRSS13 | 11 | 117779008 | 117779008 | G | A | UTR3 | . | 0.0006 | rs186475723 |
| SCN2B | 11 | 118037415 | 118037415 | C | A | UTR3 | . | 0.0006 | rs200598418 |
| DDX6 | 11 | 118650388 | 118650388 | G | A | exonic | synonymousSNV | 0.0041 | rs61756275 |
| POU2F3 | 11 | 120190138 | 120190143 | TTTTTT | - | UTR3 | . | 0.017 | . |
| UBASH3B | 11 | 122646936 | 122646936 | C | G | exonic | synonymousSNV | 0.0039 | rs34347341 |
| OR8B3 | 11 | 124266390 | 124266390 | A | G | exonic | synonymousSNV | 0.0229 | rs28496031 |
| FOXRED1 | 11 | 126141370 | 126141370 | A | C | exonic | nonsynonymousSNV | 0.0036 | rs148346044 |
| PRDM10 | 11 | 129771130 | 129771130 | G | A | UTR3 | . | 0.0009 | rs137866324 |
| ZBTB44 | 11 | 130103413 | 130103413 | C | T | UTR3 | . | 0.0003 | rs529519844 |
| WNK1 | 12 | 970296 | 970296 | - | A | exonic | frameshiftinsertion | 0.00007565 | rs746484082 |
| ADIPOR2 | 12 | 1897160 | 1897160 | A | C | UTR3 | . | 0.0046 | rs147038940 |
| ITFG2 | 12 | 2921816 | 2921816 | G | A | UTR5 | . | 0.0009 | rs193236623 |
| TSPAN9 | 12 | 3393388 | 3393388 | T | C | UTR3 | . | 0.0017 | rs71577827 |
| KCNA6 | 12 | 4923830 | 4923830 | C | T | UTR3 | . | 0.0028 | rs191635410 |
| MRPL51 | 12 | 6601593 | 6601593 | G | - | exonic | frameshiftdeletion | . | rs748824105 |
| C3AR1 | 12 | 8212776 | 8212776 | C | T | exonic | synonymousSNV | 0.0004 | rs140906071 |
| CLEC2D | 12 | 9849143 | 9849144 | GT | - | UTR3 | . | 0.0018 | . |
| CLEC7A | 12 | 10282803 | 10282803 | G | A | UTR5 | . | 0.0004 | rs775236528 |
| PYROXD1,RECQL | 12 | 21622621 | 21622621 | - | T | UTR3 | . | . | . |
| PPFIBP1 | 12 | 27846120 | 27846120 | C | T | UTR3 | . | 0.00003186 | rs993785827 |
| H3F3C | 12 | 31944476 | 31944476 | G | A | UTR3 | . | 0.0091 | rs112907660 |
| PKP2 | 12 | 33031309 | 33031309 | T | C | exonic | nonsynonymousSNV | 0.0006 | rs139139859 |
| SLC2A13 | 12 | 40152594 | 40152594 | - | T | UTR3 | . | 0.0017 | rs968572213 |
| ANO6 | 12 | 45782052 | 45782052 | G | A | exonic | nonsynonymousSNV | 0.0012 | rs142960268 |
| SLC48A1 | 12 | 48175330 | 48175330 | A | T | UTR3 | . | 0.0093 | rs117708888 |
| TUBA1B | 12 | 49525295 | 49525295 | C | A | UTR5 | . | 0.0025 | rs573614978 |
| TUBA1A | 12 | 49582819 | 49582819 | C | T | UTR5 | . | 0.0021 | rs182661252 |
| ASIC1 | 12 | 50467532 | 50467532 | C | A | exonic | synonymousSNV | 0.0059 | rs142317660 |
| LIMA1 | 12 | 50570668 | 50570676 | TGTGTTTTT | - | UTR3 | . | 0.0111 | rs201225828 |
| SLC4A8 | 12 | 51844673 | 51844673 | G | A | exonic | synonymousSNV | 0.0003 | rs146534549 |
| SLC4A8 | 12 | 51907914 | 51907914 | T | C | UTR3 | . | 0.0001 | . |
| ACVR1B | 12 | 52389284 | 52389284 | C | A | UTR3 | . | 0.0042 | rs2714 |
| KRT80 | 12 | 52564717 | 52564717 | G | A | UTR3 | . | 0.0088 | rs117183164 |
| KRT82 | 12 | 52799923 | 52799923 | G | A | exonic | stopgain | 0.0022 | rs148453926 |
| SPRYD3 | 12 | 53458912 | 53458912 | C | T | UTR3 | . | 0.0042 | rs148697931 |
| IGFBP6 | 12 | 53494940 | 53494940 | G | A | exonic | nonsynonymousSNV | 0.00003184 | rs375501582 |
| SP7 | 12 | 53720388 | 53720388 | A | - | UTR3 | . | 0.0025 | rs377039350 |
| NCKAP1L | 12 | 54911376 | 54911376 | C | T | exonic | synonymousSNV | 0.0069 | rs34286028 |
| DNAJC14 | 12 | 56215116 | 56215116 | G | A | UTR3 | . | 0.0125 | rs79015557 |
| MMP19 | 12 | 56230897 | 56230897 | C | T | exonic | nonsynonymousSNV | 0.0009 | rs145965552 |
| NACA | 12 | 57114113 | 57114113 | C | T | exonic | nonsynonymousSNV | 0.0054 | rs149200196 |
| MYO1A | 12 | 57441459 | 57441459 | G | A | exonic | stopgain | 0.0023 | rs121909305 |
| GNS | 12 | 65108762 | 65108762 | T | C | UTR3 | . | 0.0002 | rs540594590 |
| LOC100129940 | 12 | 66251834 | 66251834 | C | T | UTR3 | . | 0.0069 | rs117520590 |
| HMGA2 | 12 | 66358254 | 66358254 | C | T | UTR3 | . | 0.00006379 | rs148192099 |
| MYBPC1 | 12 | 101988756 | 101988756 | C | T | UTR5 | . | 0.0071 | rs78173516 |
| APPL2 | 12 | 105591593 | 105591593 | A | G | exonic | synonymousSNV | 0.0019 | rs140225839 |
| GLTP | 12 | 110290197 | 110290197 | G | A | UTR3 | . | 0.0022 | rs932205130 |
| GLTP | 12 | 110293565 | 110293565 | G | T | exonic | synonymousSNV | 0.0056 | rs139982828 |
| NAA25 | 12 | 112465127 | 112465127 | A | - | UTR3 | . | 0.0002 | rs202157443 |
| TESC | 12 | 117476742 | 117476742 | T | - | UTR3 | . | 0.0823 | rs543223333 |
| HCAR3 | 12 | 123199966 | 123199966 | - | AA | UTR3 | . | 0.0349 | rs748966922 |
| TCTN2 | 12 | 124172609 | 124172609 | A | G | exonic | nonsynonymousSNV | 0.0005 | rs151318349 |
| DNAH10 | 12 | 124297874 | 124297874 | A | G | exonic | nonsynonymousSNV | 0.007 | rs148503528 |
| TMEM132D | 12 | 129694074 | 129694074 | G | A | exonic | synonymousSNV | 0.0017 | rs145352969 |
| RIMBP2 | 12 | 130926715 | 130926715 | C | T | exonic | synonymousSNV | 0.0019 | rs138793493 |
| TEX26 | 13 | 31526799 | 31526799 | A | - | exonic | frameshiftdeletion | 0.0000319 | rs761757966 |
| TNFSF11 | 13 | 43181995 | 43181995 | - | T | UTR3 | . | 0.0017 | rs200198391 |
| KPNA3 | 13 | 50274834 | 50274834 | C | A | UTR3 | . | 0.0002 | rs767692632 |
| BORA | 13 | 73320856 | 73320856 | A | G | exonic | synonymousSNV | 0.0064 | rs41286054 |
| PIBF1 | 13 | 73369613 | 73369613 | G | A | exonic | nonsynonymousSNV | 0.00003186 | rs376737770 |
| LMO7 | 13 | 76334893 | 76334893 | A | G | UTR5 | . | 0.0051 | rs73227964 |
| KCTD12 | 13 | 77455197 | 77455197 | A | G | UTR3 | . | 0.00003187 | rs934468593 |
| SCEL | 13 | 78192153 | 78192153 | A | G | exonic | nonsynonymousSNV | 0.0046 | rs145663548 |
| RNF219 | 13 | 79213134 | 79213134 | T | G | exonic | nonsynonymousSNV | 0.001 | rs144588084 |
| GPC5 | 13 | 92345964 | 92345964 | G | A | exonic | synonymousSNV | 0.0004 | rs147274494 |
| PCCA | 13 | 100955204 | 100955204 | A | G | exonic | synonymousSNV | 0.0024 | rs41281120 |
| TPP2 | 13 | 103281904 | 103281904 | T | C | exonic | synonymousSNV | 0.00003184 | . |
| CCDC168 | 13 | 103396135 | 103396135 | C | T | exonic | synonymousSNV | 0.0099 | rs113303771 |
| NOVA1 | 14 | 26915658 | 26915658 | G | A | UTR3 | . | 0.0004 | rs183069455 |
| SCFD1 | 14 | 31099759 | 31099759 | T | C | exonic | nonsynonymousSNV | 0.0036 | rs61754480 |
| NUBPL | 14 | 32257065 | 32257065 | A | C | exonic | nonsynonymousSNV | 0.006 | rs11558436 |
| NKX2-8 | 14 | 37050547 | 37050547 | C | T | exonic | nonsynonymousSNV | 0.0039 | rs10135525 |
| FANCM | 14 | 45667921 | 45667921 | C | T | exonic | stopgain | 0.001 | rs144567652 |
| SOCS4 | 14 | 55512748 | 55512748 | - | T | UTR3 | . | . | . |
| PELI2 | 14 | 56764795 | 56764795 | C | T | UTR3 | . | 0.0107 | rs55731113 |
| C14orf105 | 14 | 57938118 | 57938118 | C | T | exonic | synonymousSNV | 0.0028 | rs34152531 |
| ACTR10 | 14 | 58686421 | 58686421 | C | T | exonic | nonsynonymousSNV | 0.0002 | rs370351893 |
| ARID4A | 14 | 58839281 | 58839281 | T | C | UTR3 | . | 0.0076 | rs114355066 |
| WDR89 | 14 | 64066373 | 64066373 | G | A | exonic | synonymousSNV | 0.0026 | rs752646441 |
| WDR89 | 14 | 64066379 | 64066379 | C | T | exonic | synonymousSNV | 0.003 | rs117229690 |
| WDR89 | 14 | 64066394 | 64066394 | A | G | exonic | synonymousSNV | 0.0033 | rs959084766 |
| WDR89 | 14 | 64066395 | 64066395 | C | T | exonic | nonsynonymousSNV | 0.0032 | rs992348595 |
| WDR89 | 14 | 64066398 | 64066398 | G | C | exonic | nonsynonymousSNV | 0.0032 | rs918064794 |
| WDR89 | 14 | 64066402 | 64066402 | A | G | exonic | nonsynonymousSNV | 0.0027 | rs940317302 |
| SGPP1 | 14 | 64194042 | 64194042 | G | A | exonic | synonymousSNV | 0.0007 | rs146370444 |
| SYNE2 | 14 | 64518503 | 64518503 | A | C | exonic | nonsynonymousSNV | 0.0023 | rs140325055 |
| SYNE2 | 14 | 64554434 | 64554434 | A | G | exonic | nonsynonymousSNV | 0.0018 | rs140243093 |
| ZBTB25 | 14 | 64971922 | 64971922 | - | A | UTR5 | . | 0.0085 | rs200099822 |
| SPTB | 14 | 65259994 | 65259994 | C | T | exonic | nonsynonymousSNV | . | rs779141940 |
| SPTB | 14 | 65260485 | 65260485 | T | C | exonic | synonymousSNV | 0.0018 | rs74056006 |
| ZFYVE26 | 14 | 68229060 | 68229060 | C | T | exonic | nonsynonymousSNV | 0.0077 | rs140540720 |
| ERH | 14 | 69865015 | 69865015 | A | T | UTR5 | . | 0.0028 | rs543758111 |
| PLEKHD1 | 14 | 69966901 | 69966901 | T | C | exonic | synonymousSNV | 0.00006376 | rs61745939 |
| RGS6 | 14 | 73029288 | 73029288 | G | A | UTR3 | . | 0.0056 | rs72547272 |
| PAPLN | 14 | 73729387 | 73729387 | C | T | exonic | nonsynonymousSNV | 0.0005 | rs201201042 |
| ELMSAN1 | 14 | 74183578 | 74183578 | C | T | UTR3 | . | 0.0059 | rs117259255 |
| ALDH6A1 | 14 | 74535660 | 74535660 | G | A | exonic | nonsynonymousSNV | 0.0034 | rs139579994 |
| VRTN | 14 | 74823810 | 74823810 | C | T | exonic | synonymousSNV | 0.0000319 | rs138593269 |
| YLPM1 | 14 | 75266265 | 75266265 | C | T | exonic | nonsynonymousSNV | 0.00006371 | rs369089869 |
| MLH3 | 14 | 75516351 | 75516351 | T | C | exonic | nonsynonymousSNV | 0.0081 | rs114829239 |
| TTLL5 | 14 | 76349172 | 76349172 | G | T | exonic | nonsynonymousSNV | 0.0079 | rs10130991 |
| IFT43 | 14 | 76525554 | 76525554 | C | T | UTR3 | . | 0.0035 | rs181028040 |
| VIPAS39 | 14 | 77893973 | 77893973 | G | A | exonic | stopgain | 0.00006371 | rs777254676 |
| SEL1L | 14 | 81938771 | 81938771 | A | G | UTR3 | . | 0.001 | rs373065670 |
| SEL1L | 14 | 81964326 | 81964326 | C | T | exonic | synonymousSNV | 0.001 | rs145651689 |
| DDX24 | 14 | 94526720 | 94526720 | C | T | exonic | nonsynonymousSNV | . | rs771455114 |
| SERPINA10 | 14 | 94756503 | 94756503 | G | T | exonic | nonsynonymousSNV | 0.0016 | rs147367426 |
| BDKRB1 | 14 | 96730262 | 96730262 | C | G | exonic | synonymousSNV | 0.0033 | rs140482892 |
| GOLGA8J | 15 | 30385099 | 30385099 | C | T | exonic | nonsynonymousSNV | 0.0039 | rs200523284 |
| TMCO5A | 15 | 38243475 | 38243475 | G | C | UTR3 | . | 0.001 | rs372111310 |
| PAK6 | 15 | 40558401 | 40558401 | C | G | exonic | nonsynonymousSNV | 0.00006371 | rs368829109 |
| MGA | 15 | 42058958 | 42058958 | A | C | exonic | nonsynonymousSNV | 0.0025 | rs199779997 |
| CAPN3 | 15 | 42702187 | 42702187 | C | T | exonic | synonymousSNV | 0.00006371 | rs371577901 |
| TGM7 | 15 | 43569068 | 43569068 | T | A | exonic | synonymousSNV | 0.0044 | rs150374715 |
| LCMT2 | 15 | 43622441 | 43622441 | C | A | exonic | nonsynonymousSNV | 0.0048 | rs200429843 |
| MAP1A | 15 | 43814963 | 43814963 | A | G | exonic | nonsynonymousSNV | 0.0034 | rs193113360 |
| MAP1A | 15 | 43815020 | 43815020 | A | G | exonic | nonsynonymousSNV | 0.00006374 | rs773865133 |
| CTDSPL2 | 15 | 44776467 | 44776467 | A | G | exonic | nonsynonymousSNV | 0.00009558 | rs757364972 |
| SEMA6D | 15 | 48066258 | 48066258 | G | C | UTR3 | . | 0.0225 | rs11528 |
| HDC | 15 | 50534966 | 50534966 | T | C | exonic | nonsynonymousSNV | 0.0041 | rs145672878 |
| DMXL2 | 15 | 51790979 | 51790979 | T | C | exonic | nonsynonymousSNV | 0.0076 | rs35349640 |
| CGNL1 | 15 | 57815782 | 57815782 | C | T | exonic | synonymousSNV | 0.0077 | rs150883802 |
| ICE2 | 15 | 60715526 | 60715526 | C | A | UTR3 | . | 0.0076 | rs117085279 |
| TPM1 | 15 | 63336291 | 63336291 | C | T | exonic | synonymousSNV | 0.0022 | rs17850194 |
| PLEKHO2 | 15 | 65157901 | 65157901 | G | A | exonic | synonymousSNV | 0.0098 | rs116174314 |
| PLEKHO2 | 15 | 65159270 | 65159270 | C | T | UTR3 | . | 0.0081 | rs77764243 |
| SMAD3 | 15 | 67487275 | 67487275 | T | C | UTR3 | . | 0.0111 | rs72661162 |
| CALML4 | 15 | 68484061 | 68484061 | G | A | UTR3 | . | 0.0022 | rs143358063 |
| ITGA11 | 15 | 68643659 | 68643659 | G | A | exonic | synonymousSNV | 0.0071 | rs61729770 |
| ARID3B | 15 | 74836290 | 74836292 | CAG | - | exonic | nonframeshiftdeletion | 0.0002 | rs762405564 |
| RCN2 | 15 | 77242389 | 77242389 | T | - | UTR3 | . | 0.0004 | rs577533640 |
| BTBD1 | 15 | 83686105 | 83686105 | G | A | UTR3 | . | 0.0154 | rs140540874 |
| TPSB2 | 16 | 1278478 | 1278478 | T | C | UTR3 | . | 0.0059 | rs11548897 |
| TPSD1 | 16 | 1306889 | 1306889 | G | A | exonic | nonsynonymousSNV | 0.00006371 | rs530280660 |
| TPSD1 | 16 | 1306923 | 1306923 | C | T | exonic | nonsynonymousSNV | 0.0077 | rs143993373 |
| PKD1 | 16 | 2150211 | 2150211 | G | A | exonic | nonsynonymousSNV | 0.00003186 | rs202227418 |
| TBC1D24 | 16 | 2552618 | 2552618 | G | A | UTR3 | . | 0.0062 | rs115543208 |
| MEFV | 16 | 3293369 | 3293369 | C | T | exonic | synonymousSNV | 0.0079 | rs2234939 |
| CREBBP | 16 | 3779594 | 3779594 | C | T | exonic | synonymousSNV | 0.0094 | rs61754523 |
| ZC3H7A | 16 | 11850150 | 11850150 | C | G | exonic | nonsynonymousSNV | 0.0106 | rs139825983 |
| BFAR | 16 | 14755781 | 14755781 | A | G | exonic | synonymousSNV | 0.0024 | rs146402847 |
| PDXDC1 | 16 | 15100301 | 15100301 | C | T | exonic | nonsynonymousSNV | 0.0022 | rs141901522 |
| MYH11 | 16 | 15808876 | 15808876 | C | G | exonic | nonsynonymousSNV | 0.0046 | rs113964173 |
| ABCC6 | 16 | 16259579 | 16259579 | G | A | exonic | synonymousSNV | 0.0047 | rs60975032 |
| XYLT1 | 16 | 17232375 | 17232375 | G | A | exonic | nonsynonymousSNV | . | rs199714091 |
| EEF2K | 16 | 22291670 | 22291670 | G | A | exonic | nonsynonymousSNV | 0.0005 | rs146761211 |
| USP31 | 16 | 23073702 | 23073703 | TA | - | UTR3 | . | 0.0239 | rs748536998 |
| NPIPB6 | 16 | 28354119 | 28354119 | A | G | exonic | nonsynonymousSNV | 0.0033 | rs759966996 |
| C16orf92,FAM57B | 16 | 30035806 | 30035806 | C | T | UTR3 | . | 0.0273 | rs72791208 |
| SEPT1 | 16 | 30393171 | 30393171 | C | T | exonic | nonsynonymousSNV | 0.0003 | rs41292380 |
| FBRS | 16 | 30681594 | 30681594 | C | G | UTR3 | . | 0.00003191 | . |
| SRCAP | 16 | 30749084 | 30749084 | T | A | exonic | nonsynonymousSNV | 0.0002 | rs368587038 |
| SRCAP | 16 | 30751101 | 30751101 | A | G | UTR3 | . | 0.0075 | rs192066047 |
| TGFB1I1 | 16 | 31487813 | 31487813 | C | T | exonic | synonymousSNV | 0.0043 | rs148448987 |
| ABCC11 | 16 | 48249131 | 48249131 | T | C | exonic | nonsynonymousSNV | 0.0002 | rs141417535 |
| N4BP1 | 16 | 48594876 | 48594876 | T | C | exonic | nonsynonymousSNV | 0.0002 | rs200650848 |
| PAPD5 | 16 | 50263942 | 50263942 | G | A | UTR3 | . | 0.008 | rs9931139 |
| CYLD | 16 | 50833197 | 50833197 | T | C | UTR3 | . | 0.00006376 | rs986086042 |
| CIAPIN1 | 16 | 57462928 | 57462928 | C | - | UTR3 | . | 0.0181 | rs547843808 |
| CDH5 | 16 | 66438108 | 66438108 | G | A | UTR3 | . | 0.0036 | rs140666675 |
| BEAN1 | 16 | 66511560 | 66511560 | C | T | exonic | synonymousSNV | 0.0022 | rs142343073 |
| CKLF | 16 | 66599860 | 66599860 | T | C | exonic | synonymousSNV | 0.0002 | rs368934751 |
| PLEKHG4 | 16 | 67319293 | 67319293 | G | C | exonic | nonsynonymousSNV | . | . |
| ENKD1 | 16 | 67698938 | 67698938 | C | T | exonic | synonymousSNV | 0.005 | rs115961528 |
| RANBP10 | 16 | 67759875 | 67759875 | - | TA | UTR3 | . | 0.0002 | rs575080906 |
| SLC7A6,SLC7A6OS | 16 | 68334930 | 68334930 | - | T | UTR3 | . | 0.0004 | rs904540749 |
| PRMT7 | 16 | 68371390 | 68371390 | C | A | exonic | synonymousSNV | 0.0014 | rs72792215 |
| SMPD3 | 16 | 68392371 | 68392371 | T | C | UTR3 | . | 0.00006367 | rs999377912 |
| NFAT5 | 16 | 69731364 | 69731364 | A | - | UTR3 | . | 0.0008 | rs879292301 |
| MAF | 16 | 79629022 | 79629022 | - | A | UTR3 | . | . | . |
| OSGIN1 | 16 | 83999537 | 83999537 | C | T | exonic | synonymousSNV | 0.0032 | rs117872227 |
| TAF1C | 16 | 84212875 | 84212875 | G | A | exonic | nonsynonymousSNV | 0.0025 | rs199976567 |
| PELP1 | 17 | 4577884 | 4577884 | C | G | exonic | synonymousSNV | 0.0094 | rs76038822 |
| C17orf107 | 17 | 4804691 | 4804691 | C | G | UTR3 | . | 0.0059 | rs193239361 |
| NCOR1 | 17 | 15973774 | 15973774 | T | G | exonic | nonsynonymousSNV | 0.0104 | rs61753150 |
| CCDC144NL | 17 | 20799179 | 20799179 | C | G | exonic | nonsynonymousSNV | 0.0008 | rs201978220 |
| NLK | 17 | 26521949 | 26521949 | A | - | UTR3 | . | 0.022 | rs34676938 |
| FLOT2 | 17 | 27206528 | 27206528 | C | T | UTR3 | . | 0.0087 | rs9617 |
| MYO18A | 17 | 27493950 | 27493950 | G | A | exonic | synonymousSNV | 0.0045 | rs34478330 |
| TAOK1 | 17 | 27878023 | 27878023 | A | - | UTR3 | . | 0.0008 | . |
| TP53I13 | 17 | 27896342 | 27896342 | C | G | exonic | nonsynonymousSNV | 0.0069 | rs112563021 |
| GOSR1 | 17 | 28853644 | 28853644 | G | A | UTR3 | . | 0.0082 | rs144926598 |
| RAB11FIP4 | 17 | 29864834 | 29864834 | C | T | UTR3 | . | 0.0279 | rs111605159 |
| CDK5R1 | 17 | 30815805 | 30815805 | A | C | UTR3 | . | 0.00006373 | rs756857205 |
| ASIC2 | 17 | 31340464 | 31340464 | G | C | UTR3 | . | 0.0313 | rs62068265 |
| AATF | 17 | 35414143 | 35414143 | G | A | UTR3 | . | 0.0086 | rs150772606 |
| KRTAP9-3 | 17 | 39389690 | 39389690 | G | T | UTR3 | . | 0.0661 | rs78005298 |
| HOXB1 | 17 | 46607113 | 46607113 | C | T | exonic | synonymousSNV | 0.0037 | rs35115415 |
| UBE2Z | 17 | 47004761 | 47004761 | - | C | UTR3 | . | 0.005 | rs371315163 |
| ZNF652 | 17 | 47395288 | 47395288 | C | T | UTR5 | . | 0.0193 | rs8082083 |
| NXPH3 | 17 | 47656639 | 47656639 | A | T | exonic | nonsynonymousSNV | . | rs763317845 |
| SPOP | 17 | 47676876 | 47676876 | - | G | UTR3 | . | 0.042 | . |
| DLX4 | 17 | 48050307 | 48050307 | C | A | UTR5 | . | 0.0127 | rs139488744 |
| XYLT2 | 17 | 48437932 | 48437932 | C | T | UTR3 | . | . | rs141916726 |
| MRPL27 | 17 | 48445451 | 48445451 | C | T | UTR3 | . | 0.0035 | rs138569619 |
| CHAD | 17 | 48542381 | 48542381 | G | T | UTR3 | . | 0.0034 | rs149022149 |
| UTP18 | 17 | 49337996 | 49337996 | G | A | exonic | synonymousSNV | . | . |
| STXBP4 | 17 | 53046176 | 53046176 | G | T | UTR5 | . | 0.0148 | rs17817968 |
| SRSF1 | 17 | 56082055 | 56082055 | A | G | UTR3 | . | 0.0034 | rs192838949 |
| PPM1E | 17 | 57060352 | 57060352 | A | - | UTR3 | . | 0.0002 | rs1056025556 |
| METTL2A | 17 | 60526142 | 60526142 | - | A | UTR3 | . | 0.0003 | rs373258331 |
| METTL2A | 17 | 60526799 | 60526799 | C | T | UTR3 | . | 0.0175 | rs148839698 |
| MAP3K3 | 17 | 61771631 | 61771631 | T | C | UTR3 | . | 0.0098 | rs111454356 |
| MAP3K3 | 17 | 61773158 | 61773158 | A | G | UTR3 | . | 0.0099 | rs77562968 |
| LIMD2,MAP3K3 | 17 | 61773472 | 61773472 | A | T | UTR3 | . | . | . |
| GH2 | 17 | 61958228 | 61958228 | C | G | exonic | nonsynonymousSNV | 0.00003189 | rs201550585 |
| TEX2 | 17 | 62225874 | 62225874 | T | C | UTR3 | . | 0.0172 | rs117162915 |
| DDX5 | 17 | 62499163 | 62499163 | C | T | exonic | synonymousSNV | 0.0076 | rs56025355 |
| GNA13 | 17 | 63049698 | 63049698 | T | C | exonic | synonymousSNV | 0.0081 | rs41300618 |
| APOH | 17 | 64210599 | 64210599 | G | T | exonic | synonymousSNV | 0.0102 | rs150652035 |
| BPTF | 17 | 65979140 | 65979140 | A | T | UTR3 | . | 0.0298 | rs117037971 |
| C17orf58 | 17 | 65989037 | 65989037 | A | G | exonic | stoploss | 0.0056 | rs190313223 |
| ABCA10 | 17 | 67161107 | 67161107 | G | C | exonic | nonsynonymousSNV | 0.0018 | rs138716621 |
| MTCL1 | 18 | 8793070 | 8793070 | C | G | exonic | synonymousSNV | 0.0029 | rs116633163 |
| CEP192 | 18 | 13068921 | 13068921 | C | T | exonic | synonymousSNV | 0.0011 | rs143041966 |
| ROCK1 | 18 | 18691298 | 18691298 | G | A | UTR5 | . | 0.0067 | rs112006560 |
| PSMA8 | 18 | 23731897 | 23731897 | A | G | exonic | nonsynonymousSNV | 0.0046 | rs150332181 |
| DSC3 | 18 | 28574150 | 28574150 | T | C | exonic | synonymousSNV | 0.0099 | rs114143100 |
| CCDC178 | 18 | 30913312 | 30913312 | A | G | exonic | synonymousSNV | . | . |
| DTNA | 18 | 32471180 | 32471180 | A | - | UTR3 | . | 0.001 | rs1048882009 |
| CELF4 | 18 | 34824546 | 34824546 | - | T | UTR3 | . | 0.0045 | rs200979856 |
| SETBP1 | 18 | 42531926 | 42531928 | ACA | - | exonic | nonframeshiftdeletion | 0.00003187 | rs755486319 |
| LOXHD1 | 18 | 44137400 | 44137400 | C | T | exonic | nonsynonymousSNV | 0.0129 | rs118174674 |
| CPLX4 | 18 | 56985739 | 56985739 | - | AA | UTR5 | . | 0.0037 | rs373891365 |
| SERPINB5 | 18 | 61171242 | 61171242 | G | T | UTR3 | . | 0.0032 | rs186059840 |
| FAM69C | 18 | 72114068 | 72114068 | C | T | exonic | nonsynonymousSNV | 0.00003192 | rs752901203 |
| ZADH2 | 18 | 72921027 | 72921027 | G | A | UTR5 | . | 0.0002 | rs749215833 |
| MIDN | 19 | 1259080 | 1259080 | - | A | UTR3 | . | . | . |
| DAZAP1 | 19 | 1435275 | 1435275 | T | A | UTR3 | . | . | . |
| ABHD17A | 19 | 1877023 | 1877023 | T | C | UTR3 | . | 0.0065 | rs541571053 |
| ZNF556 | 19 | 2877719 | 2877719 | G | A | exonic | nonsynonymousSNV | 0.0009 | rs144505131 |
| ANKRD24 | 19 | 4207293 | 4207293 | T | C | exonic | nonsynonymousSNV | 0.0056 | rs199801260 |
| TNFAIP8L1 | 19 | 4653086 | 4653086 | G | A | UTR3 | . | 0.0215 | rs186595311 |
| SAFB2 | 19 | 5590371 | 5590371 | C | T | exonic | nonsynonymousSNV | 0.0074 | rs150472516 |
| FBN3 | 19 | 8186254 | 8186254 | G | A | exonic | synonymousSNV | 0.0049 | rs117616097 |
| FBN3 | 19 | 8186255 | 8186255 | C | A | exonic | nonsynonymousSNV | 0.005 | rs117880701 |
| ZNF426 | 19 | 9639710 | 9639710 | T | C | exonic | synonymousSNV | 0.004 | rs45489098 |
| EIF3G | 19 | 10229580 | 10229580 | T | C | exonic | synonymousSNV | 0.0042 | rs34658893 |
| EIF3G | 19 | 10230596 | 10230596 | A | C | UTR5 | . | 0.0071 | rs191367288 |
| SMARCA4 | 19 | 11172819 | 11172819 | T | C | UTR3 | . | 0.0014 | rs146968124 |
| DOCK6 | 19 | 11327760 | 11327760 | C | T | exonic | nonsynonymousSNV | 0.0007 | rs200042437 |
| DOCK6 | 19 | 11333488 | 11333488 | C | T | exonic | nonsynonymousSNV | 0.0012 | rs202209921 |
| ZNF491 | 19 | 11919131 | 11919131 | A | T | UTR3 | . | 0.0011 | rs547272824 |
| ZNF799 | 19 | 12501081 | 12501081 | G | T | UTR3 | . | 0.0042 | rs201019469 |
| ZNF799 | 19 | 12501094 | 12501094 | G | A | UTR3 | . | 0.0007 | rs201936838 |
| ZNF443 | 19 | 12551832 | 12551832 | C | A | UTR5 | . | 0.0236 | rs117371426 |
| MAN2B1 | 19 | 12757392 | 12757392 | C | T | UTR3 | . | 0.0069 | rs149101136 |
| BRD4 | 19 | 15383794 | 15383794 | G | A | exonic | synonymousSNV | 0.0008 | rs56036027 |
| AKAP8 | 19 | 15483906 | 15483906 | C | T | exonic | nonsynonymousSNV | 0.0072 | rs111389458 |
| PGLYRP2 | 19 | 15587130 | 15587130 | C | T | exonic | synonymousSNV | 0.0001 | rs141238743 |
| OR10H2 | 19 | 15838869 | 15838869 | C | T | exonic | nonsynonymousSNV | 0.0017 | rs146389531 |
| USHBP1 | 19 | 17373761 | 17373761 | A | G | exonic | nonsynonymousSNV | 0.00003187 | rs141014235 |
| ANKLE1 | 19 | 17397493 | 17397493 | G | C | exonic | nonsynonymousSNV | . | . |
| TMEM221 | 19 | 17547222 | 17547222 | T | C | UTR3 | . | 0.0019 | rs570799335 |
| TMEM221 | 19 | 17547331 | 17547331 | T | C | exonic | nonsynonymousSNV | 0.0023 | rs191341046 |
| UNC13A | 19 | 17766944 | 17766944 | A | G | exonic | nonsynonymousSNV | 0.001 | rs200561053 |
| KIAA1683 | 19 | 18367995 | 18367995 | T | A | exonic | nonsynonymousSNV | 0.0078 | rs61740699 |
| UPF1 | 19 | 18971194 | 18971194 | C | T | exonic | synonymousSNV | 0.0076 | rs45505499 |
| MEF2BNB | 19 | 19293252 | 19293252 | G | A | UTR3 | . | 0.018 | rs17215305 |
| CILP2 | 19 | 19654762 | 19654762 | C | T | exonic | nonsynonymousSNV | 0.00009554 | rs142051343 |
| ZNF506 | 19 | 19905116 | 19905116 | T | G | UTR3 | . | . | . |
| ZNF486 | 19 | 20308149 | 20308149 | C | G | exonic | stopgain | 0.0045 | rs184976796 |
| ZNF486 | 19 | 20308488 | 20308488 | G | T | exonic | synonymousSNV | 0.0046 | rs181719180 |
| ZNF430 | 19 | 21241254 | 21241255 | AA | - | UTR3 | . | . | . |
| ZNF493 | 19 | 21607825 | 21607825 | C | A | UTR3 | . | 0.0009 | rs139042507 |
| ZNF98 | 19 | 22574900 | 22574900 | C | T | exonic | synonymousSNV | 0.0141 | rs62118622 |
| ZNF91 | 19 | 23545525 | 23545525 | T | C | exonic | nonsynonymousSNV | 0.0016 | rs139015019 |
| CEBPG | 19 | 33873199 | 33873199 | C | T | UTR3 | . | 0.0173 | rs14093 |
| KIAA0355 | 19 | 34832866 | 34832866 | C | T | exonic | nonsynonymousSNV | . | rs757904234 |
| CD22 | 19 | 35837725 | 35837725 | A | G | UTR3 | . | 0.0085 | rs73927992 |
| GAPDHS | 19 | 36036001 | 36036001 | T | C | exonic | nonsynonymousSNV | 0.0017 | rs139696096 |
| UPK1A | 19 | 36164140 | 36164140 | G | C | exonic | nonsynonymousSNV | 0.0012 | rs144711603 |
| ACTN4 | 19 | 39220802 | 39220802 | C | - | UTR3 | . | 0.0077 | rs563858750 |
| ACTN4,CAPN12 | 19 | 39221070 | 39221070 | - | T | UTR3 | . | 0.0053 | . |
| FCGBP | 19 | 40398136 | 40398136 | C | T | exonic | synonymousSNV | 0.0027 | rs587697705 |
| AKT2 | 19 | 40739522 | 40739522 | C | T | UTR3 | . | 0.0011 | rs140411527 |
| CYP2B6 | 19 | 41518598 | 41518598 | T | A | exonic | nonsynonymousSNV | 0.0042 | rs35979566 |
| PSG3 | 19 | 43244488 | 43244488 | C | T | exonic | nonsynonymousSNV | 0.0003 | rs144627053 |
| PSG1 | 19 | 43371484 | 43371484 | G | T | UTR3 | . | 0.0122 | rs117147176 |
| PSG6 | 19 | 43407828 | 43407828 | A | G | exonic | nonsynonymousSNV | 0.00003228 | rs745744148 |
| PSG6 | 19 | 43420425 | 43420425 | G | T | exonic | synonymousSNV | 0.002 | rs144161671 |
| PSG4 | 19 | 43697292 | 43697292 | G | A | UTR3 | . | 0.0005 | rs549353981 |
| PSG4 | 19 | 43709742 | 43709742 | G | T | UTR5 | . | 0.0122 | rs143507318 |
| RSPH6A | 19 | 46299165 | 46299167 | CCT | - | exonic | nonframeshiftdeletion | 0.00003228 | rs767608497 |
| SYMPK | 19 | 46318759 | 46318771 | CCCCGCCCCGTCC | - | UTR3 | . | 0.0735 | rs140334478 |
| IGFL1 | 19 | 46733560 | 46733560 | T | G | exonic | nonsynonymousSNV | 0.0051 | rs187146043 |
| LIG1 | 19 | 48640902 | 48640902 | G | A | exonic | synonymousSNV | 0.009 | rs4987069 |
| KDELR1 | 19 | 48887611 | 48887611 | C | T | exonic | synonymousSNV | 0.0003 | rs142773187 |
| SPACA4 | 19 | 49110221 | 49110221 | C | T | UTR5 | . | 0.0098 | rs186844167 |
| FUT2 | 19 | 49207687 | 49207699 | AAAAAAAAAAAAG | - | UTR3 | . | 0.0022 | . |
| CGB5 | 19 | 49547358 | 49547358 | A | G | UTR5 | . | 0.0233 | rs767891521 |
| RBBP9 | 20 | 18470558 | 18470558 | G | A | exonic | synonymousSNV | 0.00006375 | rs565219087 |
| CD93 | 20 | 23065584 | 23065584 | C | T | exonic | nonsynonymousSNV | 0.0051 | rs144703062 |
| NOL4L | 20 | 31035020 | 31035020 | T | C | UTR3 | . | 0.0003 | rs374921459 |
| COMMD7 | 20 | 31291140 | 31291140 | G | A | UTR3 | . | 0.00009558 | rs577463814 |
| EPB41L1 | 20 | 34785935 | 34785935 | C | G | exonic | nonsynonymousSNV | 0.0047 | rs6089016 |
| ARFGEF2 | 20 | 47649897 | 47649897 | A | G | UTR3 | . | 0.00003183 | rs759606916 |
| PTPN1 | 20 | 49197873 | 49197873 | C | T | exonic | nonsynonymousSNV | 0.004 | rs16995309 |
| PARD6B | 20 | 49369613 | 49369613 | - | C | UTR3 | . | 0.0006 | . |
| MX1 | 21 | 42830691 | 42830692 | CT | - | exonic | frameshiftdeletion | 0.00009615 | rs754265226 |
| HIRA | 22 | 19344558 | 19344558 | C | T | exonic | nonsynonymousSNV | 0.00009585 | rs199804204 |
| TRMT2A | 22 | 20104636 | 20104636 | C | T | UTR5 | . | 0.00009569 | rs1039093963 |
| RIMBP3 | 22 | 20457485 | 20457485 | T | C | exonic | nonsynonymousSNV | 0.0874 | rs2242315 |
| RIMBP3 | 22 | 20457514 | 20457514 | T | G | exonic | nonsynonymousSNV | 0.0683 | rs200584390 |
| RIMBP3 | 22 | 20458830 | 20458830 | C | T | exonic | synonymousSNV | 0.0163 | rs879495919 |
| KLHL22 | 22 | 20796597 | 20796597 | G | A | exonic | synonymousSNV | 0.0013 | rs145958380 |
| MED15 | 22 | 20941012 | 20941012 | C | G | UTR3 | . | 0.0012 | rs12484193 |
| SNAP29 | 22 | 21213632 | 21213632 | C | G | exonic | synonymousSNV | 0.0021 | rs144160898 |
| HIC2 | 22 | 21804135 | 21804135 | C | T | UTR3 | . | 0.0027 | rs142295205 |
| TOP3B | 22 | 22316911 | 22316911 | C | T | exonic | nonsynonymousSNV | 0.0021 | rs146766833 |
| ZNF280B | 22 | 22839428 | 22839428 | T | C | UTR3 | . | 0.0099 | rs73156169 |
| BCR | 22 | 23658813 | 23658813 | C | T | UTR3 | . | 0.0048 | rs369607920 |
| BCR | 22 | 23658996 | 23658996 | T | C | UTR3 | . | 0.0095 | rs370383730 |
| CRYBB3 | 22 | 25603245 | 25603245 | C | G | UTR3 | . | 0.0127 | rs79515519 |
| LRP5L | 22 | 25750647 | 25750647 | T | C | exonic | nonsynonymousSNV | . | rs757801653 |
| TTC28 | 22 | 28501414 | 28501414 | C | T | exonic | nonsynonymousSNV | 0.0082 | rs77885044 |
| SLC35E4 | 22 | 31032918 | 31032918 | C | G | exonic | nonsynonymousSNV | . | . |
| OSBP2 | 22 | 31303305 | 31303306 | TA | - | UTR3 | . | 0.0004 | rs1056020936 |
| DEPDC5 | 22 | 32257404 | 32257404 | C | T | exonic | synonymousSNV | 0.0002 | rs371969641 |
| MYH9 | 22 | 36745146 | 36745146 | G | A | exonic | nonsynonymousSNV | 0.0072 | rs147122501 |
| TXN2 | 22 | 36863247 | 36863247 | A | C | UTR3 | . | 0.0361 | rs73169629 |
| TXN2 | 22 | 36863675 | 36863676 | CT | - | UTR3 | . | 0.0359 | rs148887681 |
| TMEM184B | 22 | 38616340 | 38616340 | G | A | UTR3 | . | 0.0002 | rs756668258 |
| TNRC6B | 22 | 40727675 | 40727675 | - | A | UTR3 | . | 0.07 | rs776164567 |
| PHF5A | 22 | 41864700 | 41864700 | G | C | UTR5 | . | 0.0042 | rs112844862 |
| POLR3H | 22 | 41922489 | 41922489 | G | - | UTR3 | . | 0.0047 | rs548509220 |
| BIK | 22 | 43525331 | 43525331 | G | A | UTR3 | . | 0.0002 | rs201603625 |
| PKDREJ | 22 | 46654159 | 46654159 | T | C | exonic | synonymousSNV | 0.009 | rs147904316 |
| CELSR1 | 22 | 46758735 | 46758735 | G | A | UTR3 | . | 0.0089 | rs55642103 |
| FTH1P18 | X | 37061592 | 37061592 | G | T | exonic | nonsynonymousSNV | . | . |
| PRRG1 | X | 37300968 | 37300968 | G | A | UTR3 | . | 0.0034 | rs149902784 |
| DMRTC1,DMRTC1B | X | 72092282 | 72092282 | G | A | UTR3 | . | 0.0003 | rs782532391 |
| POF1B | X | 84569510 | 84569510 | C | T | exonic | synonymousSNV | 0.0004 | rs141403678 |
| SYTL4 | X | 99930926 | 99930926 | T | C | UTR3 | . | . | rs868529234 |
| HNRNPH2 | X | 100663294 | 100663294 | C | T | UTR5 | . | 0.0000923 | rs781968054 |
| NUP62CL | X | 106418405 | 106418405 | G | A | UTR5 | . | 0.0009 | rs201593300 |
| HTR2C | X | 114144547 | 114144547 | T | A | UTR3 | . | 0.0075 | rs111873124 |
| SLC6A14 | X | 115582750 | 115582750 | C | T | exonic | synonymousSNV | 0.0062 | rs12720085 |
| GRIA3 | X | 122624669 | 122624669 | G | A | UTR3 | . | 0.0116 | rs34568700 |
| RAB33A | X | 129305916 | 129305917 | AC | - | UTR5 | . | . | . |
| IGSF1 | X | 130412680 | 130412680 | T | G | exonic | nonsynonymousSNV | 0.0087 | rs146462069 |
| HS6ST2 | X | 132092608 | 132092608 | A | - | exonic | frameshiftdeletion | 0.002 | rs750023244 |
